# Concave and Convex Molecular Curvature Modulates Spatial Electronic Environments for Controlled Electrocatalysis

**DOI:** 10.1021/jacs.6c01391

**Published:** 2026-06-13

**Authors:** Fuping Pan, Yang Cheng, Jian Cai, Yun Song, Yinger Xin, Jianjun Su, Haoyang Li, Maoyu Wang, Ting Wang, Yuexiang Hou, Ruquan Ye, Kai-Jie Chen

**Affiliations:** † School of Chemistry and Chemical Engineering, Northwestern Polytechnical University, Xi′an, Shaanxi 710072, China; ‡ Department of Chemistry and State Key Laboratory of Marine Environmental Health, City University of Hong Kong, Kowloon, Hong Kong 999077, China; § Shanghai Synchrotron Radiation Facility, Shanghai Advanced Research Institute, Chinese Academy of Sciences, Shanghai 201204, China

## Abstract

Molecular catalysts offer well-defined active sites and tailorable structures that together govern their intrinsic activity. While molecular curvature has emerged as a powerful tool for modulating catalytic activity, the role of the local curvature environment remains poorly understood. Here, we demonstrate that the catalytic properties of iron phthalocyanines (FePc) are strongly influenced by the spatial concave and convex architectures. Although FePc has been predominantly reported to catalyze four-electron O_2_ reduction, reports of its two-electron pathway are rare. By depositing FePc on carbon supports with a cylindrical mesopore (concave-FePc) and its inverse architecture (convex-FePc), we demonstrate that convex-FePc preferentially catalyzes the four-electron O_2_ reduction route, whereas concave-FePc favors the two-electron pathway, primarily producing H_2_O_2_ with selectivity exceeding 80%. In situ electrochemical infrared spectroscopy and theoretical calculations reveal that local concave/convex geometries of FePc modulate the electronic properties of the Fe site and its interaction with key intermediates via spatial orbital rearrangement. Specifically, the confined environment under the concave curvature reduces orbital overlaps between Fe *d*_z2_ of FePc and O *p* of *OOH, thereby weakening *OOH adsorption and boosting O_2_-to-H_2_O_2_ conversion. This curvature-dependent activity also extends to CoPc/MnPc and electrochemical CO_2_ reduction, underscoring the versatility of this approach. Our findings present a general design framework for engineering the catalytic performance of molecular catalysts through a tailored curvature environment.

## Graphical abstract

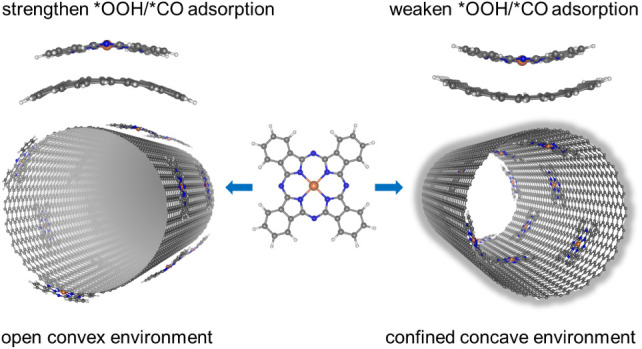

## Introduction

Oxygen reduction reaction (ORR) plays a critical role in chemical and electrical energy conversion. Molecular O_2_ can either undergo a four-electron (O_2_ + 4H^+^ + 4e^–^ → 2H_2_O) or two-electron (O_2_ + 2H^+^ + 2e^–^ → H_2_O_2_) reduction pathway.^1–4^ The former is the cathodic reaction in fuel cells,^5^ while the latter offers a sustainable route toward the production of H_2_O_2_, a vital chemical currently synthesized via an energy-intensive thermochemical anthraquinone approach.^6^ Both of the two ORR pathways share the same intermediate of *OOH from O_2_ activation. A strong *OOH binding promotes the cleavage of the O–O bond to generate O* and OH* intermediates associated with the complete 4e^–^ reduction, whereas moderately weakened adsorption retains the O–O bond, resulting in the evolution of H_2_O_2_.^7–9^ To fulfill the requirement of different application scenarios, it is of great significance to develop an effective strategy for finely tuning *OOH binding strength and ORR pathways on heterogeneous catalysts.

Carbon-supported single-atom catalysts (SACs) have emerged as promising alternatives to the platinum group metal catalysts for ORR.^1,3,6,10,11^ Of particular interest, organometallic molecular catalysts, such as metallophthalocyanines (MPc) with M–N_4_ moiety, provide a model SAC platform for studying binding behaviors of intermediates on single-metal sites because of their well-defined atomic configuration and tunable electronic properties.^12–18^ For plane M–N_4_ sites with strong *OOH binding, they typically yield a 4e^–^ ORR route, while weak *OOH binding favors the 2e^–^ pathway. Structural deformation engineering to tailor geometric microenvironments has been proposed to modulate the electronic structure and adjust the adsorption strength of intermediates in electrocatalytic processes.^3,19–24^ For instance, convex carbon topology has been demonstrated to influence the ORR selectivity of metal-free N-doped carbon.^25^ For carbon-supported single-metal sites, supporting single-atom Pt on onion-like carbon with high curvature can induce a local electric field, accelerating the catalytic kinetics in the hydrogen evolution reaction.^26^ Compared to planar FePc, curved FePc supported on carbon nanotubes promotes the 4e^–^ ORR pathway due to the strain effect,^27^ and curved CoPc was also found to improve *CO binding and thus facilitate deep CO_2_ reduction into methanol.^20^

The structural distortion reported hitherto was mainly introduced by locating molecular complexes at the convex solid supports. This effect modulates the single-metal site’s *d*-orbital energy of molecular catalysts and creates strong *d*-*p* interactions to reinforce binding with intermediates in a spatially open environment.^20^ Alternatively, the reaction can take place on the inner surface of curved molecular catalysts when metal complexes are implanted in the concave supports.^28–30^ In such a space-confined local reaction environment, the electron redistribution of the single-metal site and its orbital hybridization with adsorbates may differ from the outer case, potentially leading to different reaction mechanisms. However, insights in engineering concave/convex electronic environments of curved molecular catalysts to adjust electrocatalytic reactivity remain poorly understood.

In this work, we study the effects of spatial environment on the electronic structure and catalytic properties of curved molecular catalysts. By supporting FePc on cylindrical ordered mesoporous carbon and its inverse counterpart, curved FePc model catalysts with exclusive concave and convex surfaces can be constructed (Figure 1), named as concave-FePc and convex-FePc, respectively. We demonstrate that concave-FePc favors 2e^–^ ORR, primarily producing H_2_O_2_, whereas convex-FePc catalyzes ORR via a 4e^–^-dominant pathway. In situ infrared spectroscopy and theoretical calculations disclose that the local environment of curved Fe–N_4_ influences spatial orbital arrangement and modulates the interaction of FePc with key intermediates. Compared to the outer environment, the confined environment of concave-FePc within the inner tube restrains Fe *d*_z2_ and O *p* orbital overlapping, thereby weakening *OOH adsorption and promoting 2e^–^ O_2_-to-H_2_O_2_ conversion. We also extend the concave/convex-tuned ORR selectivity of FePc to CoPc/MnPc and electrochemical CO_2_ reduction, suggesting the versatility of reactive environment engineering in adjusting electrocatalytic pathways of curved molecular catalysts.

**Figure 1:**
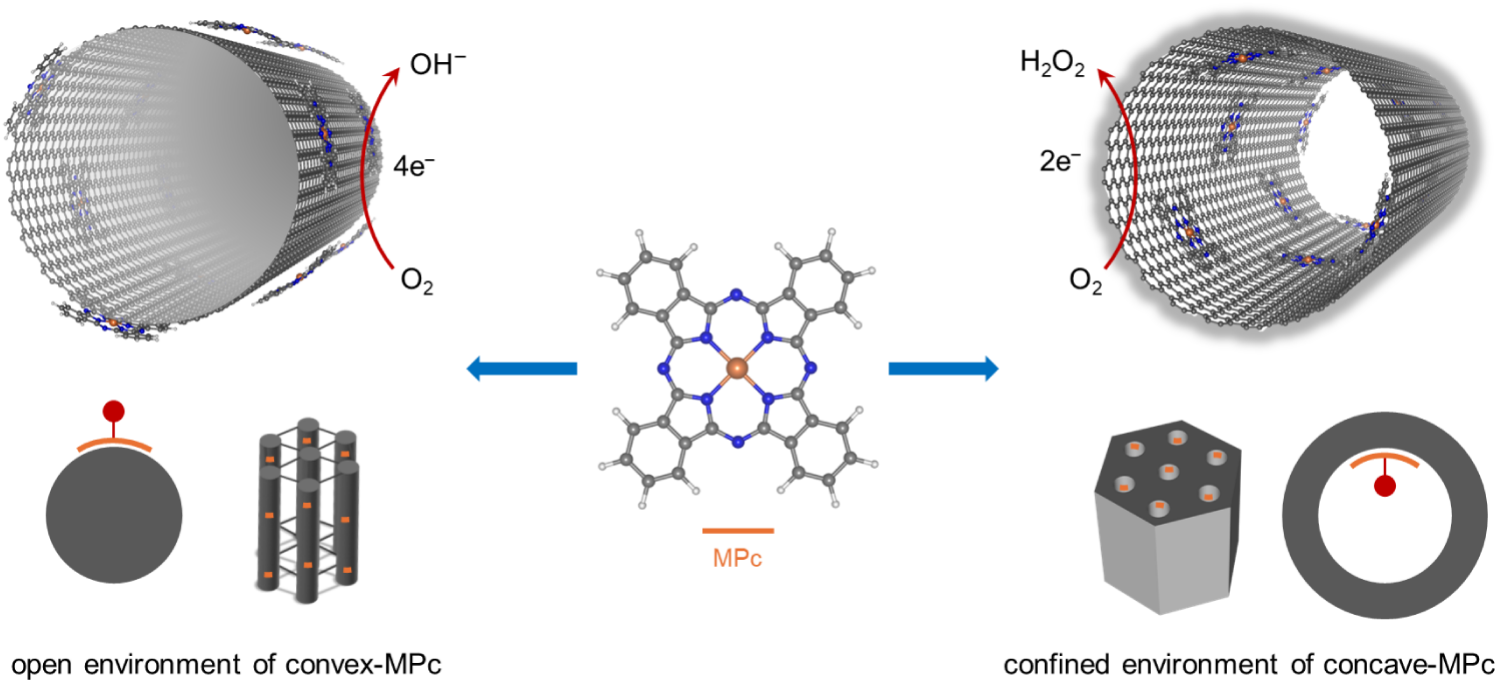
Schematic illustration of ORR pathway regulation via local curvature environment engineering. Open reactive environment of convex-MPc (left) and confined reactive environment of concave-MPc (right). The gray, blue, orange, and white balls represent C, N, M, and H atoms, respectively. The red ball refers to intermediates adsorbed on the single-metal site of MPc.

## Results and Discussion

### Design, Synthesis, and Structure of Model Catalysts

When MPc was deposited on curved carbon supports, the local π-π interactions would induce the controllable distortion of MPc molecules to match the geometric configurations of supports. Building appropriate carbon support is thus the cornerstone to investigate the effects of concave and convex curvature on electronic properties of MPc. As depicted in Figure 1, supporting MPc on cylindrical mesoporous carbon (c-MC) provides an ideal model to make the inside surface available in electrocatalytic reactions, and its inverse mesoporous carbon (i-MC) only allows the outside surface to be accessible in reactions. We synthesized c-MC by a soft-template method via the self-assembly of resol and high-temperature carbonization.^31^ Subsequent deposition of FePc yields the concave-FePc model catalyst. For convex-FePc, mesoporous silica (SBA-15) that preserves similar mesostructure parameters to c-MC was used as a hard template to direct i-MC synthesis (Figures S1, S2), and FePc was then loaded on i-MC using a similar wet-chemical deposition.

Transmission electron microscopy (TEM) images reveal that concave-FePc exhibits highly ordered cylindrical and strip-like mesopores along (100) and (110) directions embedded in the bulk of carbon (Figure 2a). The mesostructure of convex-FePc is an inverse replication of the mesoporous silica template (Figure 2b), and scanning TEM energy-dispersive spectroscopy (STEM-EDS) mapping discloses a uniform distribution of Fe and N throughout the carbon support (Figure 2c). Raman spectra indicate that the two carbon supports carbonized at the same temperature show similar D and G peaks (Figure S3). The ordered mesostructure can be confirmed by small-angle X-ray diffraction (SXRD), in which the well-resolved (100) peak is a reflection of the two-dimensional hexagonal space group of *P*6*mm* (Figure 2d).^32^ The SXRD (100) peak of concave-FePc positions at a smaller angle than that of convex-FePc, suggesting the larger unit cell of concave-FePc. N_2_ adsorption–desorption isotherms were collected to evaluate surface areas and porosities (Figure S4). The Brunauer–Emmett–Teller (BET) surface area of convex-FePc was determined to be 985.5 m^2^ g^–1^, larger than that of concave-FePc (703.1 m^2^ g^–1^). The pore size distribution profiles exhibit a well-defined peak at 3.18 and 3.64 nm for concave-FePc and convex-FePc (Figure 2e), respectively. The former results from the hexagonally arranged micelles that are assembled from F127 and resol, while the latter is a consequence of the removal of the silica template. As illustrated in Figure S5, both the pore size of concave-FePc and the rod diameter of convex-FePc are 3.2 nm. Given the lateral dimension of MPc is approximately 1.3 nm, carbon supports with a diameter of around 1 to 15 nm are commonly used to induce viable curvature.^20,29,33^ The sizes of i-MC and c-MC are thus small enough to induce local convex and concave curvature through the strong π-stacking interaction between MPc and the carbon support that can provide stabilization force to make the deformation of MPc energetically feasible.

**Figure 2:**
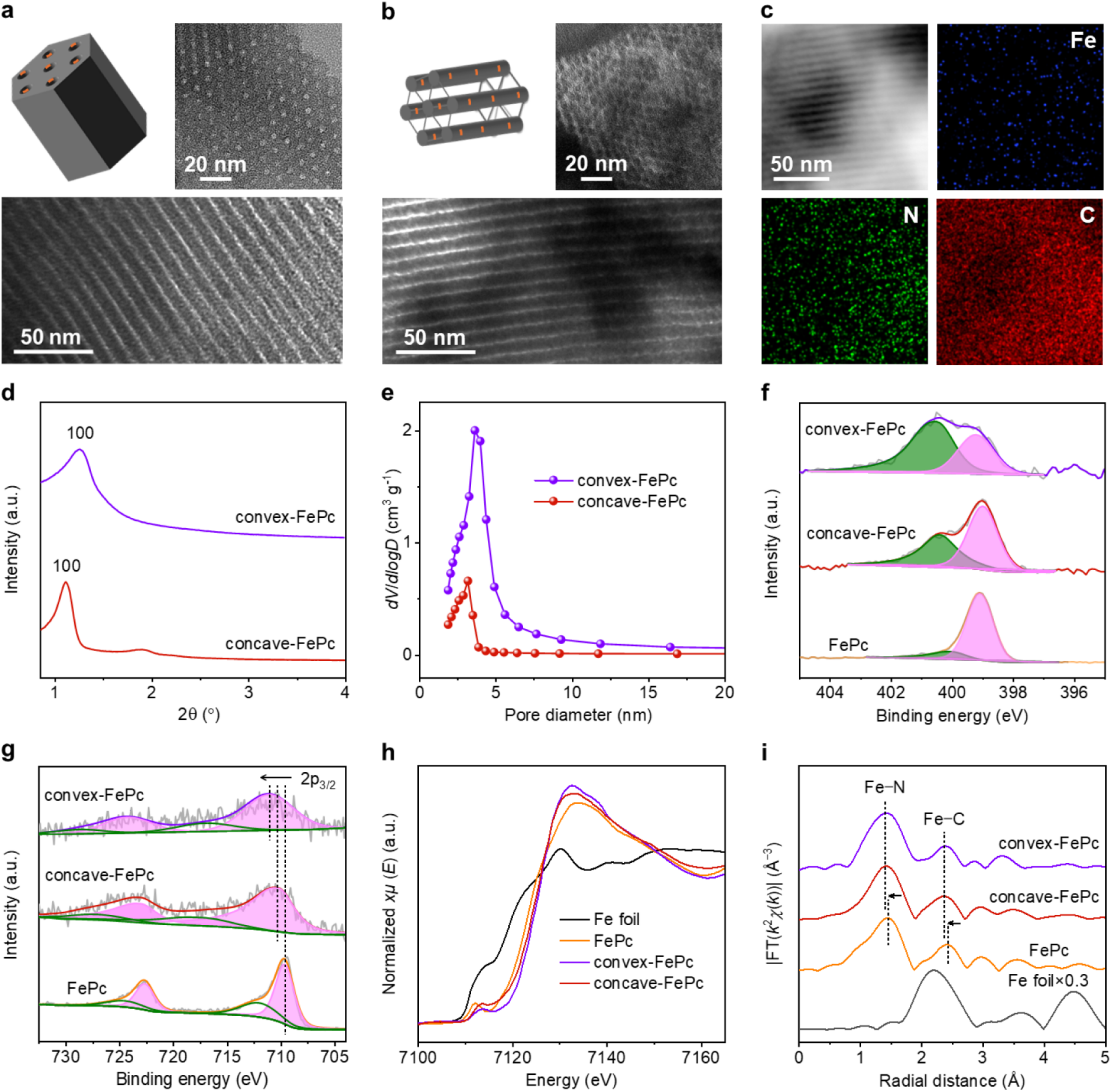
Structural and electronic properties of catalysts. (a) Scheme and TEM images of concave-FePc. (b) Scheme and TEM images of convex-FePc. TEM images in (a) and (b) are viewed along the (100) direction (upper panel) and (110) direction (bottom panel). (c) EDS elemental mapping images of convex-FePc. (d) Small-angle XRD patterns and (e) pore size distributions for convex-FePc and concave-FePc. (f) XPS N 1s, (g) XPS Fe 2p, (h) Fe K-edge XANES, and (i) Fourier transform EXAFS spectra of convex-FePc, concave-FePc, and FePc reference.

### Electronic Properties of Concave and Convex FePc

The effect of local concave and convex environments on the chemical state of FePc was studied by X-ray photoelectron spectroscopy (XPS) with standard FePc as a reference. Figure 2f shows the N 1s XPS spectra, and atomic contents were summarized in Table S1. Although the phthalocyanine ring contains two types of nitrogen, their binding energies are similar.^13,34,35^ Compared to the FePc standard that displays one main single peak at 399.1 eV, we noted the peak splitting on both concave-FePc and convex-FePc with a new peak appearing at 400.5–400.6 eV, suggesting that the curved structure causes a significant change in the chemical state of Fe–N yet has a minor effect on C–N species. The observation of peak splitting indicates strong FePc-curved carbon interaction, as demonstrated in previous studies.^20^ Convex-FePc exhibits a considerably higher peak intensity at 400.6 eV than concave-FePc, implying stronger interaction between FePc and i-MC support. Moreover, the Fe 2*p* XPS spectrum of FePc displays two peaks at 709.6 and 722.7 eV (Figure 2g), assigned to 2*p*_3/2_ and 2*p*_1/2_ of Fe^2+^, respectively. A shift of the Fe 2*p* peak to higher binding energy was observed for concave-FePc and convex-FePc, which we attribute to the π-π stacking interaction between the curved FePc and carbon support.^36^ Notably, convex-FePc exhibits a more positive shift with the Fe 2*p*_3/2_ peak centered at 711.1 eV close to Fe^3+^, indicating a higher Fe oxidation state than concave-FePc. This suggests that the Fe center of convex-FePc presents a more electron-deficient state, likely a consequence of different Fe–N interactions caused by local convex and concave curvature.

The Fe electronic properties and local atomic configurations were further explored by X-ray absorption spectroscopy (XAS). The Fe K-edge X-ray absorption near-edge structure (XANES) spectrum of FePc shows a pre-edge peak at 7112.1 eV (Figure 2h), attributed to the 1*s* to 4*p*_*z*_ transition of the Fe–N_4_ square flat structure.^37^ A decline in intensity and a shift to a higher energy position were observed in both convex-FePc and concave-FePc in comparison with FePc, indicative of distortion of planar symmetry. The adsorption edge position (7121.5 eV) of convex-FePc and concave-FePc is located at a larger energy than FePc, which suggests a higher valence state of Fe and agrees with XPS results. The Fourier transform extended X-ray absorption fine structure (EXAFS) spectra show the coordination environment around the Fe site. FePc presents two peaks at 1.46 and 2.42 Å (Figure 2i), corresponding to the Fe–N and Fe–C paths, respectively. The Fe–N/C peaks of convex-FePc and concave-FePc shift toward a more negative direction relative to FePc; this indicates a decreased Fe–N/C distance in the curved FePc. Specifically, the fitting in R space demonstrates that the Fe–N/Fe–C bond length is ∼1.96/2.97 Å for FePc, which declines to ∼1.95/2.95 Å for convex-FePc and to ∼1.95/2.94 Å for concave-FePc (Figure S6, Table S2). The tiny change in the Fe–N bond length can be attributed to the structural relaxation to release molecular strain when loading flat FePc on a curved substrate, and similar phenomena have been reported on other curved systems.^3,38^ Although convex-FePc and concave-FePc have similar Fe–N/Fe–C lengths, the single Fe-atom center of convex-FePc has a higher oxidation state than that of concave-FePc. This infers that the spatially open environment imposes a different interaction between the Fe 3*d* and N 2*p* orbitals of molecular FePc from the spatially confined case.

### Electrocatalytic Pathways and Performance Evaluation

To explore whether concave/convex surface impact ORR pathways of curved molecular catalysts, electrocatalytic ORR performance of these catalysts was evaluated using a rotating ring-disk electrode (RRDE) at 1600 rpm in an O_2_-saturated 0.1 M KOH electrolyte. The collection efficiency of the ring electrode was calibrated via a one-electron reversible redox conversion of Fe­(CN)_6_^4–^/Fe­(CN)_6_^3–^ couple (Figure S7). The ring electrode was set at 1.2 V to oxidize H_2_O_2_ formed at the disk electrode, thus distinguishing 2e^–^ and 4e^–^ pathways. FePc-containing catalysts exhibit significantly larger current density and more positive onset potential than FePc-free c-MC and i-MC, confirming the occurrence of direct O_2_ reduction on FePc (Figures S8, S9). The ORR activity was optimized by adjusting the feeding mass ratios of FePc:carbon support. The current density and onset potential increase upon improving feeding mass ratios of FePc:carbon from 1 wt % to 3 wt % (Figures S10, S11), while only a slight increase in current density was observed when the feeding mass ratio was further increased to 6 wt %. As for H_2_O_2_ selectivity, a larger feeding mass ratio of FePc:carbon leads to a slight decrease in H_2_O_2_ formation. This is probably because a denser population of FePc may promote the tandem reduction of HO_2_^–^ before it transports away from the catalyst surface.

In linear sweep voltammetry (LSV) curves (Figure 3a), concave-FePc exhibits smaller disk current density yet larger ring current density than convex-FePc at the wide potential range, implying better electrocatalytic activity of concave-FePc toward H_2_O_2_ production. The number of transferred electrons (n) further demonstrates that convex-FePc prefers to catalyze ORR with a nearly 4e^–^ pathway (Figure S12), while concave-FePc is selective toward 2e^–^ O_2_ reduction. The n values derived from K-L fitting plots based on RDE tests further confirm different reaction pathways (Figures S13, S14). The maximum H_2_O_2_ selectivity of concave-FePc reaches 82.8% at 0.53 V (Figure 3b), 3-fold larger than that of convex-FePc. This finding manifests the local curvature-dependent ORR pathways of FePc catalysts. To further verify the intrinsic selectivity of the concave environment, we have prepared FePc/graphene and FePc/carbon nanotube as references. Due to the negligible local curvature, 2D graphene and large-diameter carbon nanotubes (40–50 nm) can be considered as planar surfaces. Graphene is supposed to suffer mass transport limitations in electrocatalytic reactions due to the lack of sufficient mass transport channels, while carbon nanotubes facilitate mass transport, as 1D fibers can build macroporous networks. Despite differences in morphology, surface area, pore distribution, and defect density, the two catalysts exhibit high selectivity for 4e^–^ ORR with H_2_O_2_ selectivity less than 30% (Figures S15–S18). This suggests that mass transport and porous properties are not the main factors controlling ORR selectivity, although their roles cannot be fully excluded. Instead, the electronic properties of FePc might be the decisive factor determining the ORR pathway. Together with different ORR activity of convex-FePc and concave-FePc, it can be concluded that flat and convex FePc prefer 4e^–^ ORR, while the concave environment is highly active for the 2e^–^ pathway. In literature, carbon-supported FePc molecular catalysts^12,27,39–42^ and carbon-supported Fe–N_4_ SACs^24,43^ generally show 4e^–^-dominant ORR activity (Figure 3c), and demonstration of selective 2e^–^ ORR over Fe–N_4_ sites is sporadic. Our concave-surface engineering of curved FePc breaks the performance bottleneck and achieves efficient 2e^–^ O_2_-to-H_2_O_2_ reduction on FePc molecular catalysts. These catalysts were further applied in electrochemical CO_2_ reduction. The maximum CO Faradaic efficiency (FE) decreases from 35.1% on FePc/graphene to 26.5% on convex-FePc, yet increases to 50.2% on concave-FePc (Figure S19). It is likely that the convex surface may enhance *CO adsorption on the single Fe site of FePc relative to the flat surface, leading to lower CO FEs, while the concave surface decreases *CO adsorption that improves CO FEs. These results suggest the feasibility of local curvature engineering to tune reaction pathways for other electrochemical processes, beyond ORR.

**Figure 3:**
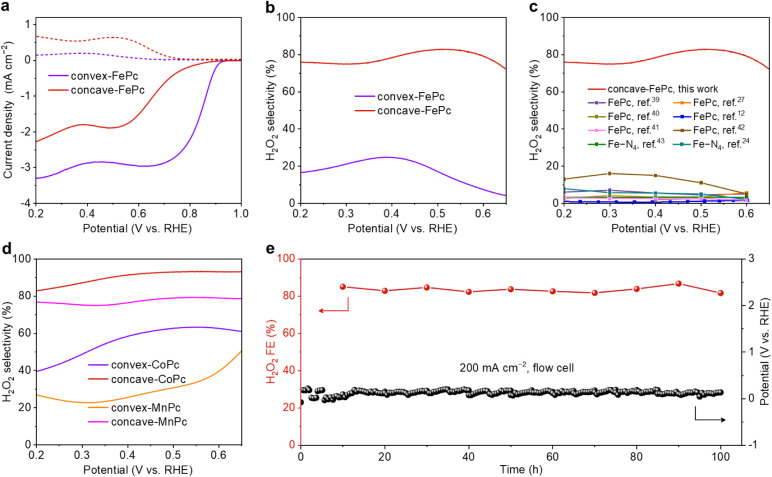
Electrocatalytic O_2_ reduction performance. (a) ORR LSV curves recorded on the disk electrode (bottom panel) and H_2_O_2_ oxidation LSV at the ring electrode (upper panel) at 1600 rpm, and (b) corresponding H_2_O_2_ selectivity for convex-FePc and concave-FePc. (c) H_2_O_2_ selectivity comparisons of reported FePc molecular catalysts, Fe–N_4_-based single-atom catalysts, and concave-FePc. (d) H_2_O_2_ selectivity for CoPc- and MnPc-based catalysts in a potential range of 0.2–0.65 V. (e) Long-term stability of concave-FePc for H_2_O_2_ electrosynthesis at 200 mA cm^–2^ in a flow cell.

We further extended this concept to CoPc and MnPc systems to verify the versatility of the local curvature engineering strategy. The N 1s and Co/Mn 2p XPS spectra of convex-CoPc­(MnPc) and concave-CoPc­(MnPc) show a similar trend to those observed on FePc-based catalysts (Figures S20, S21). In ORR, both concave-CoPc and concave-MnPc exhibit larger ring current, smaller n, and higher H_2_O_2_ selectivity than their convex-CoPc and convex-MnPc counterparts (Figure 3d, Figures S22, S23). These results strongly manifest that local curvature engineering is a general approach to tune ORR pathways on curved MPc molecules. Among the three concave-MPc catalysts, concave-CoPc shows the highest H_2_O_2_ selectivity up to 93%, and concave-FePc and concave-MnPc display similar H_2_O_2_ selectivity of ∼80%. This finding agrees with reported results that the single-atom Co center is more selective than Fe and Mn for 2e^–^ O_2_ reduction.^12,44–46^ Moreover, a three-compartment flow cell electrolyzer was employed to evaluate practical O_2_-to-H_2_O_2_ conversion by depositing concave-FePc on a hydrophobic gas-diffusion electrode to enhance O_2_ supply (Figure S24). The concentration of H_2_O_2_ was determined via a cerium titration method (Figure S25). It works stably in a 100-h continuous operation with FEs exceeding 80% at a constant current density of 200 mA cm^–2^ (Figure 3e), indicating great promise for practical electrosynthesis of H_2_O_2_ from direct O_2_ reduction.

### Mechanism Insights by ATR-SEIRAS Analyses

We applied in situ attenuated total reflection surface-enhanced infrared absorption spectroscopy (ATR-SEIRAS) to probe surface-adsorbed intermediates and their transformation during the electrocatalytic ORR process. Figure 4a, b shows ATR-SEIRAS spectra collected under open circuit potential (OCP) and ORR-related potentials ranging from 0.8 to 0.2 V vs RHE. Both convex-FePc and concave-FePc present three absorption bands at around 1455.7, 1222.5, and 1112.7 cm^–1^, which can be assigned to surface adsorbed *O_2_,^47^ *OOH,^48–51^ and *OH,^52^ respectively. The band of H_2_O_2_, typically centered at 1386 cm^–1^,^47^ was not observed, probably because HO_2_^–^ rather than H_2_O_2_ was generated in the alkaline electrolyte. The data supports that the two catalysts involve a similar ORR process with the same intermediates. Due to the high energy barrier of the direct O–O bond breakage, the surface-adsorbed *O_2_ is likely activated to *OOH, followed by the breakage of the O–O bond to form *OH, a typical associative ORR mechanism.^53–55^ No potential-dependent shift of these peaks was seen, the same as previously reported ORR-involved intermediates yet different from the *CO intermediate.^48,56^ This might be because ORR-involved intermediates have weak electronic back-donation with the catalyst surface, and there exists strong electrolyte-induced hydrogen-bonding stabilization that buffers against field-induced perturbation, rendering the bond vibration of intermediates not highly susceptible to interfacial electric field variations.^57,58^

**Figure 4:**
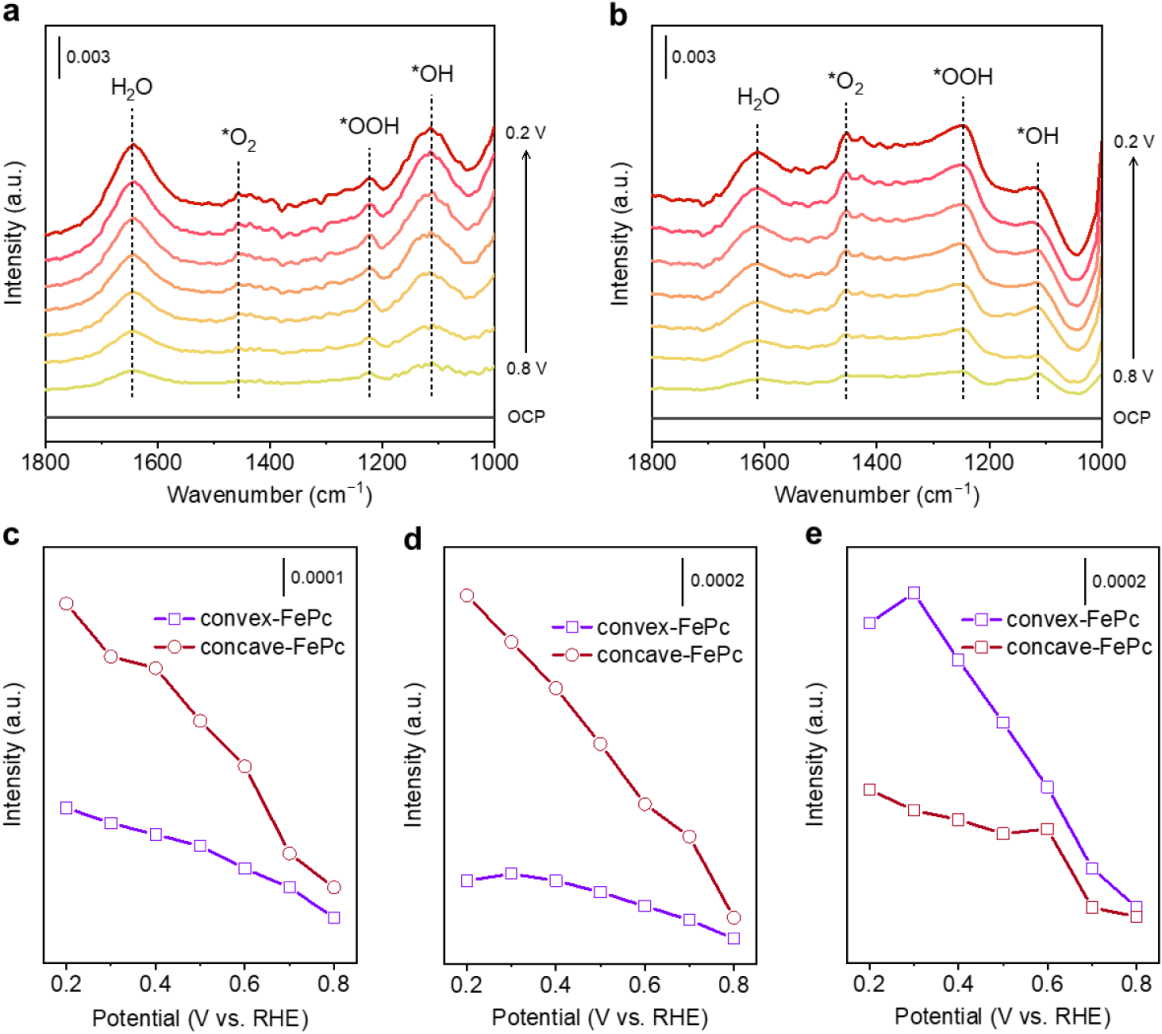
Mechanism analysis via electrochemical in situ ATR-SEIRAS. (a, b) ATR-SEIRAS spectra of convex-FePc (a) and concave-FePc (b) in the ORR process. (c–e) Band intensity of reactive species against potential for *O_2_ (c), *OOH (d), and *OH (e) on convex-FePc and concave-FePc. The signal is absorbance in ATR-SEIRAS.

The intensities of these peaks were plotted against applied potential to analyze potential-dependent transformation of surface-adsorbed species. The change in peak intensity of concave-FePc behaves differently from those of convex-FePc upon sweeping potential negatively (Figure 4c–e), hinting at different catalytic ORR mechanisms on the two catalysts. Specifically, the intensity of *O_2_ increases more slowly on convex-FePc (increasing rate of 0.0005 au/V) than that on concave-FePc (increasing rate of 0.0013 au/V) as the potential was scanned from 0.2 to 0.8 V. This indicates that convex-FePc presents faster kinetics in the protonation of *O_2_ upon increasing overpotential. For *OOH species, increasing rates of 0.0005 au/V on convex-FePc and 0.0022 au/V on concave-FePc were seen, suggesting that the formed *OOH can be more quickly transformed on convex-FePc than concave-FePc. In addition, convex-FePc presents lower *OOH intensity yet higher *OH intensity than concave-FePc, and the intensity of *OH increases more quickly on convex-FePc (increasing rate of 0.0024 au/V) than that on concave-FePc (increasing rate of 0.0009 au/V) when potentials were swept negatively. These results reflect that a large amount of *OOH is converted into *OH on convex-FePc, whereas the main portion of *OOH on concave-FePc might be reduced to HO_2_^–^ that quickly diffuses into electrolyte. This finding manifests an OOH*-mediated associative ORR pathway and the enhanced 4e^–^ ORR selectivity of convex-FePc in comparison with concave-FePc, in agreement with electrochemical ORR performance.

### DFT Calculations

The local environment-dependent ORR selectivity implies a change in the interaction with ORR-involved intermediates under concave/convex geometries of curved MPc. To explore the underlying mechanism, we applied density functional theory (DFT) calculations. A single-wall carbon nanotube (CNT) with a diameter of around 3.1 nm, similar to the pore size of c-MC and the pillar size of i-MC support, was built to mimic the reaction surface of concave-FePc and convex-FePc (Figure S26), respectively. A part of CNT with 82 carbon atoms terminated by H atoms was used as support to immobilize the FePc molecule via π-π stacking. As shown in Figure 5a, b, the flat FePc molecule spontaneously distorts to adapt to the configuration of carbon supports, showing structural deformation with obvious curvature upon supporting FePc on both the interior and exterior of the CNT due to strong π-π interactions.^27^ Based on projected density of states (pDOS) analyses (Figure S27), convex-FePc displays stronger electronic interaction between the Fe atom and adjacent N atoms than concave-FePc, as indicated by the enhanced Fe 3*d* and N 2*p* orbital overlapping of convex-FePc. We also analyzed the charge density difference (Figure S28), showing that the Fe center of convex-FePc exhibits more electron depletion than that of concave-FePc. Accordingly, convex-FePc generates a more electron-deficient Fe center than concave-FePc, in accordance with experimental XPS/XAS results.

**Figure 5:**
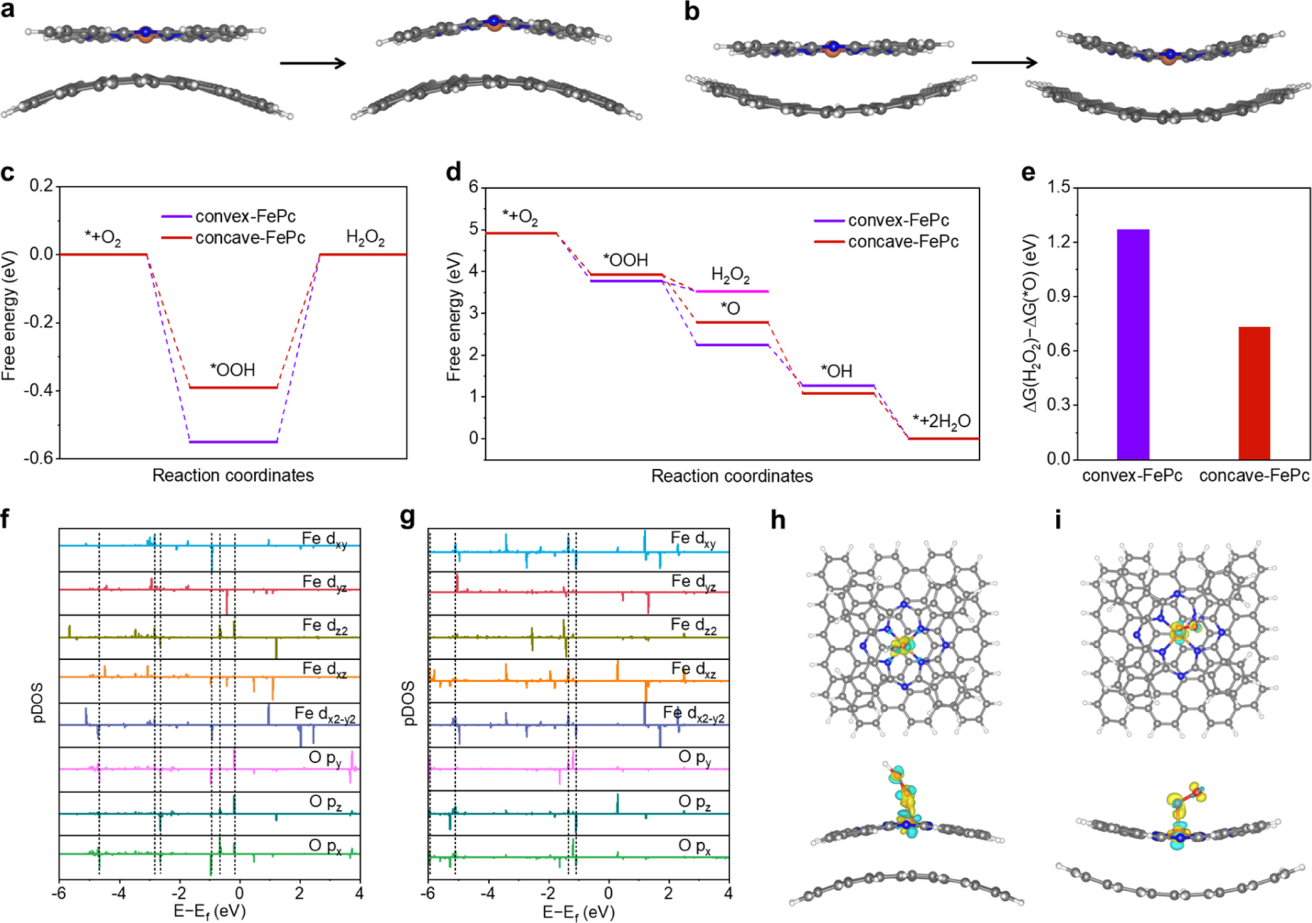
Mechanism insights by DFT calculation. (a, b) Optimized structure for convex-FePc (a) and concave-FePc (b). (c) Gibbs free energy diagrams for 2e^–^ ORR toward H_2_O_2_ formation at U = 0.6 V. (d) Gibbs free energy diagrams for 2e^–^ and 4e^–^ ORR pathways at U = 0 V. (e) Free energy difference of ΔG­(H_2_O_2_) – ΔG­(*O). (f, g) Calculated pDOS showing the orbital interaction between the Fe *d* orbitals of FePc and O *p* orbitals of *OOH for convex-FePc (f) and concave-FePc (g). (h, i) Charge density difference analysis for *OOH adsorbed on convex-FePc (h) and concave-FePc (i). Yellow and cyan isosurfaces indicate the electron accumulation and depletion. The gray, blue, orange, red, and white balls represent C, N, Fe, O, and H atoms, respectively.

We further calculated and compared the full free-energy diagram, of which the elemental pathways involving *OOH, *O, and *OH intermediates were considered. The proton-coupled electron transfer (PCET)-enabled activation of O_2_ to *OOH was employed in DFT calculations,^6,28^ and the adsorption configurations of Fe-bound intermediates were given in Figure S29. The Gibbs free energy diagrams display that the free energy barrier of H_2_O_2_ formation is 0.39 eV for concave-FePc, lower than 0.55 eV for convex-FePc (Figure 5c). The results suggest that concave-FePc is more active for 2e^–^ ORR toward H_2_O_2_ generation with weaker *OOH adsorption than convex-FePc; a similar trend was observed on CoPc- and MnPc-based systems (Figure S30). For 4e^–^ ORR, it is thermodynamically uphill at U = 1.23 V but becomes thermodynamically spontaneous at U = 0 V (Figure 5d, Figure S31). We further compared the selectivity between 2e^–^ and 4e^–^ ORR pathways. Previous studies proposed that the difference of ΔG­(H_2_O_2_) – ΔG­(*O) can serve as a descriptor to reflect the catalytic 2e^–^ ORR selectivity,^6^ of which ΔG­(H_2_O_2_) and ΔG­(*O) are defined as ΔG­(H_2_O_2_) – ΔG­(*OOH) and ΔG­(*O) – ΔG­(*OOH), respectively. A more negative value means a better 2e^–^ ORR selectivity. As depicted in Figure 5e, concave-FePc exhibits a ΔG­(H_2_O_2_) – ΔG­(*O) value of 0.73 eV, smaller than that of convex-FePc (1.27 eV), further confirming that concave-FePc is intrinsically more selective for 2e^–^ O_2_ reduction than convex-FePc.

Most M–N_4_ sites prefer the 4e^–^ ORR pathway over the 2e^–^ route, mainly ascribed to the strong adsorption of the *OOH intermediate. To boost 2e^–^ ORR, weakening the *OOH binding strength is necessary.^6,12,44^ Based on the structural characterization, we attributed the distinctive selectivity to the different local reaction environments of FePc, which influence the spatial orbital arrangement and determine adsorption strength between adsorbates and single-metal sites, as supported by the following prediction. Previous studies have demonstrated that the *d*-*p* interaction between the single-atom transition metal and surface-adsorbed intermediates mainly dictates adsorption behaviors.^59–62^ We then calculated orbital interaction between the Fe *d* orbitals of FePc and the O *p* orbitals of *OOH. In convex-FePc, the Fe-*OOH interaction is mainly triggered by *d*_x2‑y2_-*p*_*x*_/*p*_*y*_, *p*_*y*_-*d*_*xy*_/*d*_z2_, and *d*_z2_-*p*_*x*_/*p*_*y*_/*p*_*z*_ orbital hybridization below the Fermi level (Figure 5f). Among them, the Fe *d*_*yz*_ orbital does not participate in the *OOH binding, while the *d*_z2_, a high-energy orbital, plays the most significant role in binding *OOH, in agreement with reported results.^63^ By contrast, the Fe *d*_z2_ orbital does not make a contribution to the adsorption of *OOH over concave-FePc, as there is no orbital overlapping with the O *p* orbitals of *OOH (Figure 5g), thus reducing the *p*-*d* interaction and weakening *OOH adsorption on concave-FePc. Charge density difference analyses further manifest that concave-FePc presents weaker electron interaction with *OOH than convex-FePc (Figure 5h, i). In sum, our DFT results reveal that the curved FePc in the confined environment of the inner nanotube demonstrates weaker electronic interaction of Fe *d*_z2_ and O *p* orbitals and thereby reduces *OOH binding strength, which explains our experimental observation of enhanced O_2_-to-H_2_O_2_ selectivity on concave-FePc.

## Conclusions

We demonstrated the effective regulation of electrocatalytic pathways of molecular catalysts via molecular-level engineering of concave and convex curvature. We discovered that FePc with convex curvature mainly catalyzes 4e^–^ O_2_ reduction, while the concave FePc is highly selective for the 2e^–^ ORR pathway, producing H_2_O_2_ with selectivity above 80%. We revealed that the local curvature environments of curved FePc significantly influence electronic properties of the single-metal Fe site and its interaction with intermediates through spatial orbital rearrangement. The convex-FePc, at the external side of the tube with a spatially open environment, displays enhanced orbital hybridization between Fe *d*_z2_ of FePc and O *p* of *OOH, which reinforces binding with *OOH to facilitate O–O bond breakage. In contrast, concave-FePc, within the inner tube with a space-confined environment, prohibits *d*_z2_-*p* orbital interactions, which weakens *OOH adsorption and promotes selective O_2_-to-H_2_O_2_ transformation. The versatility of this conception on the regulation of ORR pathways was verified on CoPc and MnPc, both showing concave/convex curvature-dependent selectivity. The local curvature engineering was also demonstrated to tune reaction pathways in electrochemical CO_2_ reduction. While we found that curved carbon surface can induce molecular curvature and proposed that the concave/convex geometry plays a critical role in electrocatalysis, the effect of mass transport cannot be fully ruled out. This work elucidates how the spatial environments of curved molecules influence electronic structure and interaction with adsorbates, offering a general design principle to control catalytic behaviors of molecular catalysts.

## Supplementary Material



## Notes

Detailed experimental procedures, characterization methods, computational methods, and additional figures­(PDF)
