# Supplementary material for: Concave and Convex Molecular Curvature Modulates Spatial Electronic Environments for Controlled Electrocatalysis
Source: J Am Chem Soc. 2026 Jun 13;148(25):25491–500. doi: 10.1021/jacs.6c01391 (PMC13339143; doi:10.1021/jacs.6c01391)
Supplement: Supplementary file 1 [file ja6c01391_si_001.pdf]

Supporting Information

Concave and Convex Molecular Curvature Modulates Spatial Electronic Environments for Controlled Electrocatalysis

Fuping Pan,[1,#,*] Yang Cheng,[1,#] Jian Cai,[1,#] Yun Song,[2] Yinger Xin,[2] Jianjun Su,[2] Haoyang Li,[1] Maoyu Wang,[3] Ting Wang,[1] Yuexiang Hou,[1] Ruquan Ye,[2,*] Kai-Jie Chen[1,*]

[1] School of Chemistry and Chemical Engineering, Northwestern Polytechnical University, Xi'an, Shaanxi 710072, China

[2] Department of Chemistry and State Key Laboratory of Marine Environmental Health, City University of Hong Kong, Hong Kong, China

[3] Shanghai Synchrotron Radiation Facility, Shanghai Advanced Research Institute, Chinese Academy of Sciences, Shanghai, 201204 China

[#] These authors contributed equally to this work.

* Corresponding author emails: fupingpan@nwpu.edu.cn; ruquanye@cityu.edu.hk; ckjiscon@nwpu.edu.cn

Methods

Catalysts preparation

Synthesis of ordered mesoporous carbon with cylindrical mesopores (c-MC)

A solvent evaporation-induced self-assembly method was employed to synthesize c-MC. Briefly, 1 g of F127 was dissolved in a 20 g of ethanol solution. Then, 5 g of 20 wt% resol in ethanol solution was added and stirred for 30 min at room temperature to form a homogeneous solution. The mixture was transferred to Petri dishes to evaporate the solvent for 6-8 h. After heating in an oven at 100 °C for 24 h, the obtained transparent film was scrapped and crushed to obtain fine powder. Calcination was then carried out in a tubular furnace at 350 °C for 3 h and then at 900 °C for 2 h with a ramp rate of 1 °C min^{-1} under an argon atmosphere.

Synthesis of ordered mesoporous carbon with inverse architecture (i-MC)

i-MC was prepared using ordered mesoporous SiO_2 (SBA-15) as a template and sucrose as a carbon source. SBA-15 was synthesized according to the following procedures. 4 g of triblock copolymer $EO_{20}PO_{70}EO_{20}$ (P123, Mn = 5800) was dissolved in 30 g of deionized water at 35-40 °C. Subsequently, 120 g of 2 M hydrochloric acid and 8.5 g of tetraethyl orthosilicate (TEOS) was added sequentially, and the resulting mixture was stirred vigorously at room temperature for 24 h. The solution was then transferred into a polytetrafluoroethylene-lined autoclave and hydrothermally treated at 100 °C for 24 h. After the reaction, the product was separated by centrifugation, washed by deionized water, and dried at 80 °C. Finally, the dried powder was placed in a muffle furnace and calcined at 550 °C for 6 h under an air atmosphere to obtain SBA-15.

In the synthesis of i-MC, 1.25 g of sucrose and 0.14 g of H_2SO_4 were dissolved in 5 g of H_2O. Then, 1 g of as-prepared SBA-15 was added and stirred for 0.5 h at room temperature to

form a homogeneous solution. After heating at 100 °C for 6 h in an oven, the solid sample was crushed and further heated at 160 °C for 6 h. The resulting brownish black powder was dispersed into a solution of 0.5 g sucrose, 0.09 g H_2SO_4 and 5 g H_2O. After stirring for 60 min, the mixture was heated at 100 °C for 6 h and 160 °C for 6 h. The powder was annealed at 900 °C for 4 h under an argon atmosphere in a tubular furnace under. The obtained carbon/silica composite was washed by hydrofluoric acid solution (5 wt%) to remove the silica, followed by washing with H_2O and ethanol, and dried at 80 °C overnight to yield i-MC.

Synthesis of concave-MPc and convex-MPc

c-MC and i-MC were used as carbon supports to prepare concave-MPc and convex-MPc, respectively. In a typical fabrication of concave-FePc, 30 mg of c-MC was added to 20 mL DMF solution, referred as solution A. Meanwhile, a certain amount of FePc (mass ratio of FePc:c-MC is 3 wt%) was added to another DMF solution (20 mL), referred as solution B. Solution A and B were then sonicated for at least 1 h to disperse c-MC and FePc in the DMF solution. Then, solution A and B were mixed and sonicated for at least 1 h. The resulting mixture solution was vigorously stirred for 24 h at room temperature. Subsequently, the mixture was centrifuged, and the precipitate was washed with DMF, ethanol and deionized water. Finally, the precipitate was lyophilized to yield concave-FePc. convex-FePc was prepared using a method same to concave-FePc while using i-MC as the support. concave-CoPc, concave-MnPc, convex-CoPc, and convex-MnPc were synthesized via a similar method except replacing FePc by CoPc or MnPc.

Synthesis of FePc/graphene and FePc/carbon nanotube

Graphene was prepared by annealing graphene oxide, synthesized using the improved Hummers' method,[1] in H_2/Ar flow (10 vol% H_2) at 800 °C for 1 h. The carbon nanotube with

a diameter of around 40-50 nm was purchased from XFNANO, which was pretreated in 6 M HCl solution for 24 h to remove metal impurities. The graphene and purified carbon nanotube were then used as supports to load FePc via a wet-chemical deposition method similar to the synthesis of convex-FePc, and the catalysts were labeled as FePc/graphene and FePc/carbon nanotube.

Materials characterizations

The morphology, structure, and composition of catalysts were characterized by transmission electron microscopy (TEM, FEI Talos F200X), high-angle annular dark-field scanning transmission electron microscopy (HAADF-STEM, FEI Themis Z), X-ray diffraction (XRD, Bruker-D8), X-ray photoelectron spectroscopy (XPS, Kratos AXIS Ultra DLD), Raman spectroscopy (Alpha300R), and inductively coupled plasma mass spectrometer (ICP-MS, NexIONTM 350D). N_2 sorption isotherms were collected at 77 K using a specific surface area analyzer (BSD-660S). The pore size distribution was derived using Barrett-Joyner-Halenda (BJH) model. In X-ray Absorption Spectroscopy (XAS) experiments, the energy of the incident monochromatic X-rays was selected by using a water-cooled Si (111) double crystal monochromator. XAS spectra (except metal foil) were collected in the fluorescence mode, and XAS data analyses were processed with ATHENA and ARTEMIS programs.

Electrochemical measurements

The electrochemical ORR measurements were conducted in a standard three electrode system. A rotating ring disk electrode (RRDE, Pine Research Instrumentation) consisting of a glassy carbon electrode (disk area: 0.247 cm^2) and a platinum ring electrode (ring area: 0.187 cm^2) was used as the working electrode. A Hg/HgO electrode and a graphite rod were used as the reference and counter electrodes, respectively. All the recorded potentials were converted to

reversible hydrogen electrode (RHE) by $E_{RHE} = E_{Hg/HgO} + 0.114 + 0.0591 \times pH$. To prepare the working electrode, 5 mg of the catalysts and 50 μL of Nafion solution were mixed with 2 mL isopropanol and then ultrasonicated for 2 h until a homogeneous catalyst ink was obtained. After polishing the RRDE mechanically with an alumina suspension, 5 μL of catalyst ink was dropped on the disk electrode and dried to form a uniform thin film at room temperature. Electrochemical ORR performance was carried out in O_2-saturated 0.1 M KOH aqueous solution. LSV polarization curves were acquired at a scan rate of 5 mV s^{-1} and a RRDE rotation speed of 1600 rpm under O_2-saturated conditions, and the Pt ring potential was held at 1.2 V to quantify the amount of generated H_2O_2 at the disc electrode. The collection efficiency (N) of the ring electrode was calibrated to be 36.2% via a one-electron reversible redox conversion of $Fe(CN)_6^{4-}/Fe(CN)_6^{3-}$ couple. I_R and I_D are the ring current and disc current, respectively. Selectivity of H_2O_2 and electron transfer number (n) were calculated by the following equations.

$$H_2O_2\,(\%) = 200 \times \frac{I_R/N}{|I_D| + I_R/N}$$

$$n = 4 \times \frac{|I_D|}{|I_D| + I_R/N}$$

The n in ORR was also measured by RDE based on the Koutecky-Levich equation:

$$\frac{1}{I} = \frac{1}{I_L} + \frac{1}{I_K}$$

$$I_L = 0.62 \cdot F \cdot D_0^{2/3} \cdot \upsilon^{-1/6} \cdot C_0 \cdot n \cdot \omega^{1/2}$$

where I, I_L, and I_K represent measured, diffusion-limiting, and kinetic current density, F is the Faraday constant (96485 C mol^{-1}), D_0 is the diffusion coefficient of O_2 in the electrolyte (1.85×10^{-5} cm^2 s^{-1}), υ is the kinetic viscosity in the electrolyte (0.89×10^{-2} cm^2 s^{-1}), C_0 is the bulk concentration of O_2 in the electrolyte (1.21×10^{-6} mol cm^{-3}), n is the number of transferred electrons, and ω is the disk electrode angular rotation rate (rad s^{-1}). n can be

estimated from the slope of the K–L plots (I^{-1} is the y-axis, and $\omega^{-1/2}$ is the x-axis).

To achieve industrial current densities and amplify H_2O_2 concentrations, we performed the electrochemical ORR in a three-electrode flow cell electrolyzer. Catalyst ink was sprayed onto a gas diffusion layer as the cathode with a catalytic area of 1 cm^2 and a mass loading of 0.5 mg cm^{-2}. A titanium mesh loaded with IrO_2 and Hg/HgO electrode were used as the counter and reference electrodes, respectively. The cathode and anode chamber was separated by an ionic exchange membrane. 1 M KOH was used as electrolyte with a flow rate of 10 mL min^{-1} in each chamber. High-purity O_2 was purged from diffusion channel to the catalyst layer at a flow rate of 20 sccm. Long-term durability was evaluated via chronopotentiometry method at a constant current density of 200 mA cm^{-2}.

The concentration of H_2O_2 was determined by the Ce^{4+} titration method based on the stoichiometry of $2Ce^{4+} + H_2O_2 \rightarrow 2Ce^{3+} + 2H^+ + O_2$.[2] The H_2O_2 electrolyte was added into 3 mM Ce^{4+} standard solution to reduce Ce^{4+} to Ce^{3+}. The concentration of Ce^{4+} can be photometrically determined by UV-vis spectrum at the wavelength of 318 nm. The calibration curve was plotted using the known concentration of standard Ce^{4+} solution. Thus, H_2O_2 concentration and FE were calculated as the followings.

$$C\,(H_2O_2) = [V(Ce^{4+}) \times C_0(Ce^{4+}) - (V(Ce^{4+}) + V(H_2O_2)) \times C(Ce^{4+})] / (2 \times V(H_2O_2))$$

$$\mathrm{FE} = (C(H_2O_2) \times V \times 2 \times 96485) / (i \times t)$$

where $C_0(Ce^{4+})$ and $C(Ce^{4+})$ are the initial and the final concentration of Ce^{4+}, respectively, $V(Ce^{4+})$ is the volume of the Ce^{4+} standard solution, $V(H_2O_2)$ is the collected H_2O_2 electrolyte used for concentration detection, V is the volume of the electrolyte in cathodic chamber, i is the current, and t is the electrolysis time.

Electrocatalytic CO_2 reduction activity was evaluated in an H-type cell in CO_2-saturated 0.1 M $KHCO_3$ electrolyte (pH=6.8). A graphite rod and a saturated Ag/AgCl were used as the

counter electrode and reference electrode, respectively. The measured potentials were rescaled to the reversible hydrogen electrode by E (RHE) = E (Ag/AgCl) + 0.197 + 0.0591×pH. The working electrode was prepared by drop-casting catalyst ink onto a carbon paper with a catalyst loading of 0.5 mg cm^{-2}. The ink was prepared by dispersing 3 mg catalyst in a mixture solution of 200 μL DI-water, 370 μL ethanol, and 30 μL 5% Nafion solution via sonication for 4 h. The working and reference electrodes were placed in the cathode chamber, while the counter electrode was placed in the anode chamber, which was separated by a piece of Nafion 115 ionic exchange membrane to avoid the re-oxidation of CO_2RR-generated products. The high-purity CO_2 was introduced in the cathode chamber for 0.5 h to saturate electrolyte before electrolysis. The gas-phase products were analyzed via an online gas chromatograph (GC, GC9790PLUS).

FE of CO was calculated based on the equation:

$$\mathrm{FE} = \frac{z \cdot P \cdot F \cdot V \cdot v_i}{R \cdot T \cdot i}$$

Where z is the number of electrons transferred per mole of gas product (2 for CO), F is Faraday constant (96485 C mol^{-1}), P is pressure (1.01×10^5 Pa), V is the gas volumetric flow rate (5.67×10^{-7} m^3 s^{-1}), v_i is the volume concentration of gas product determined by GC, T is the temperature (298.15 K), R is the gas constant (8.314 J mol^{-1} K^{-1}), i is the steady-state current at each applied potential (A).

In situ ATR-SEIRAS measurements

In situ ATR-SEIRAS measurements were performed on a Nicolet iS50 FTIR spectrometer (Thermo Scientific) equipped with a liquid-nitrogen-cooled mercury cadmium telluride (MCT) detector. The catalyst loaded on an Au-coated Si prism was used as the working electrode, and

a graphite rod and an Hg/HgO electrode were used as the counter and reference electrodes, respectively. 0.1 M KOH was used as the electrolyte, and O_2 was continually purged at a flow rate of 40 sccm. In situ ATR-SEIRAS spectra were recorded by by 32 scans at a spectral resolution of 4 cm^{-1}, and the spectrum collected under the OCP was used as the background.

DFT calculations

The computational simulations were performed by using density functional theory as implemented in the Vienna ab initio simulation package (VASP).[3-4] The ion-electron interactions were treated with the projected augmented wave (PAW) pseudopotentials, and the plane-wave basis set was cut off at 400 eV. Generalized gradient approximation with the revised Perdew-Burke-Ernzerhof (GGA-PBE) functional was used to determine the exchange-correlation energy. The Brillouin zone was sampled by the Monkhorst-Pack method with a 2×2×1 k-point grid, and the van der Waals correction was introduced by Grimme (DFT-D3). The energy and force convergence criteria were set to be 10^{-5} eV and 0.01 eV $Å^{-1}$, respectively. H atoms-terminated 82 carbon atoms of single-wall CNT with a diameter of 3.1 nm was used as support to immobilize FePc molecule. A Hubbard-U parameter of U=5.0 eV was added to the d orbitals of Fe atom, and a combined implicit solvent method was implemented for rest of the vacuum space by the VASPsol code.

The Gibbs free energy was calculated by the following equation:

$$\Delta G = \Delta E + \Delta E_{ZPE} - T\Delta S$$

Where ΔE, ΔE_{ZPE} and ΔS denotes the change of DFT energy, zero-point energy, and entropy at 298.15 K, respectively.

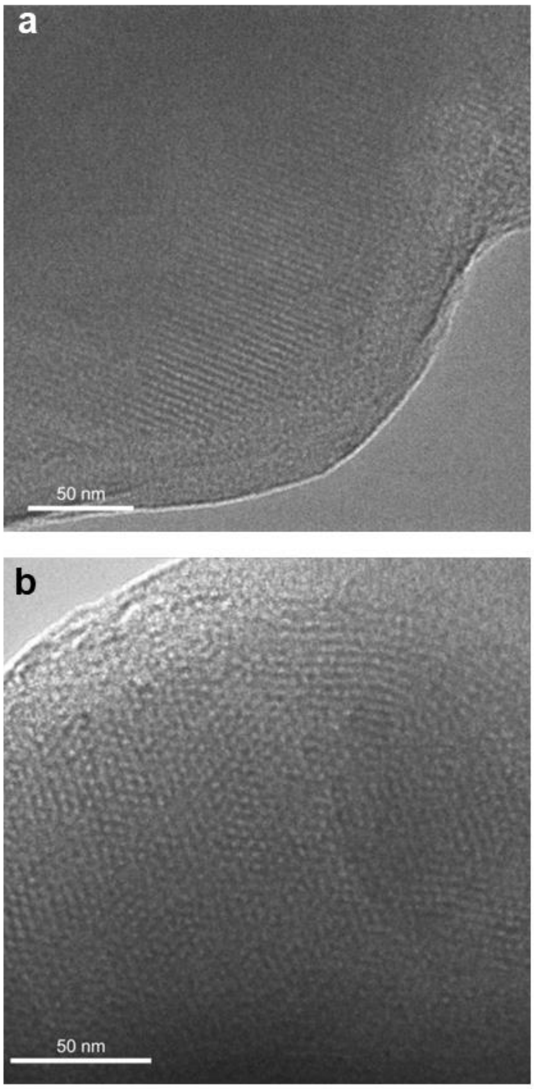

Figure S1. (a, b) TEM images of SBA-15.

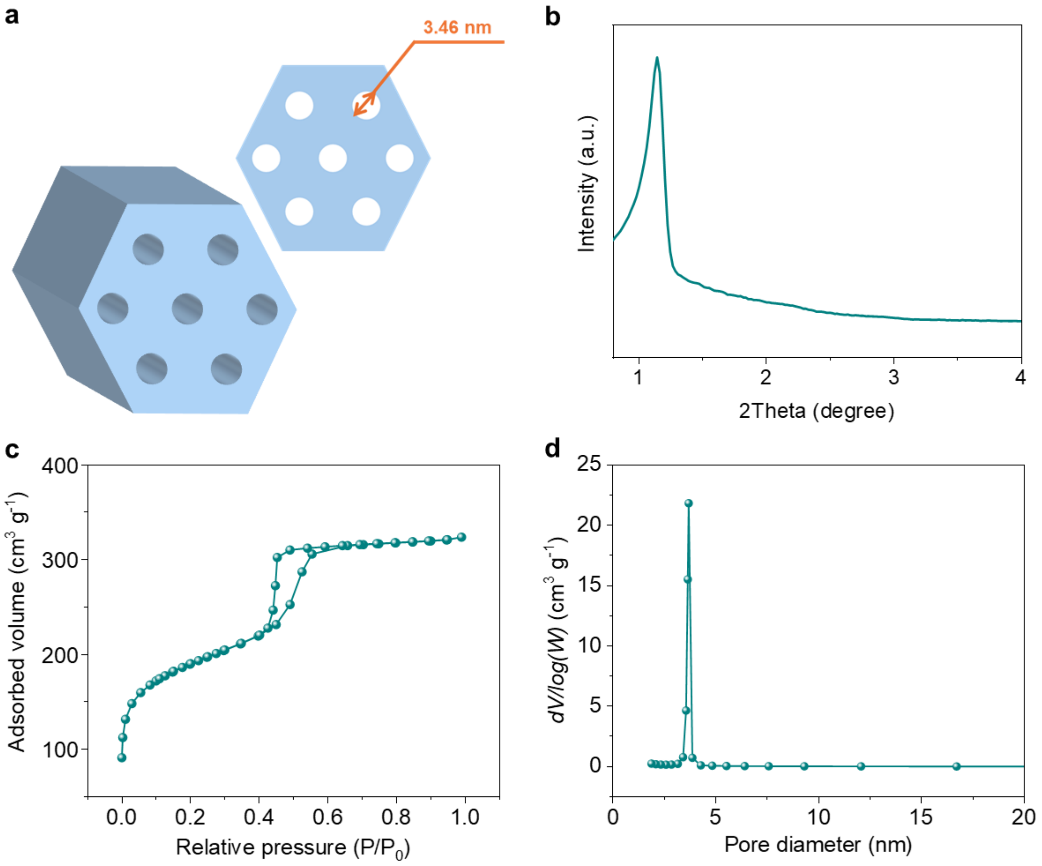

Figure S2. (a) Scheme of SBA-15. (b) Small-angle XRD pattern, (c) N_2 adsorption-desorption isotherms, and (d) pore size distribution of SBA-15.

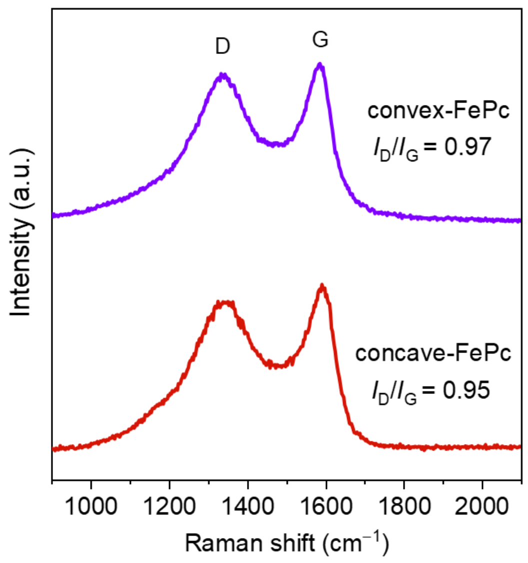

Figure S3. Raman spectra of convex-FePc and concave-FePc.

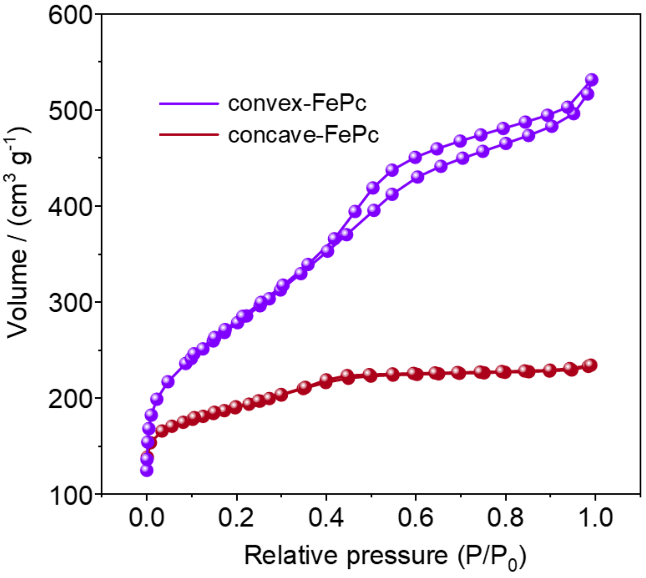

Figure S4. N_2 adsorption-desorption isotherms for convex-FePc and concave-FePc.

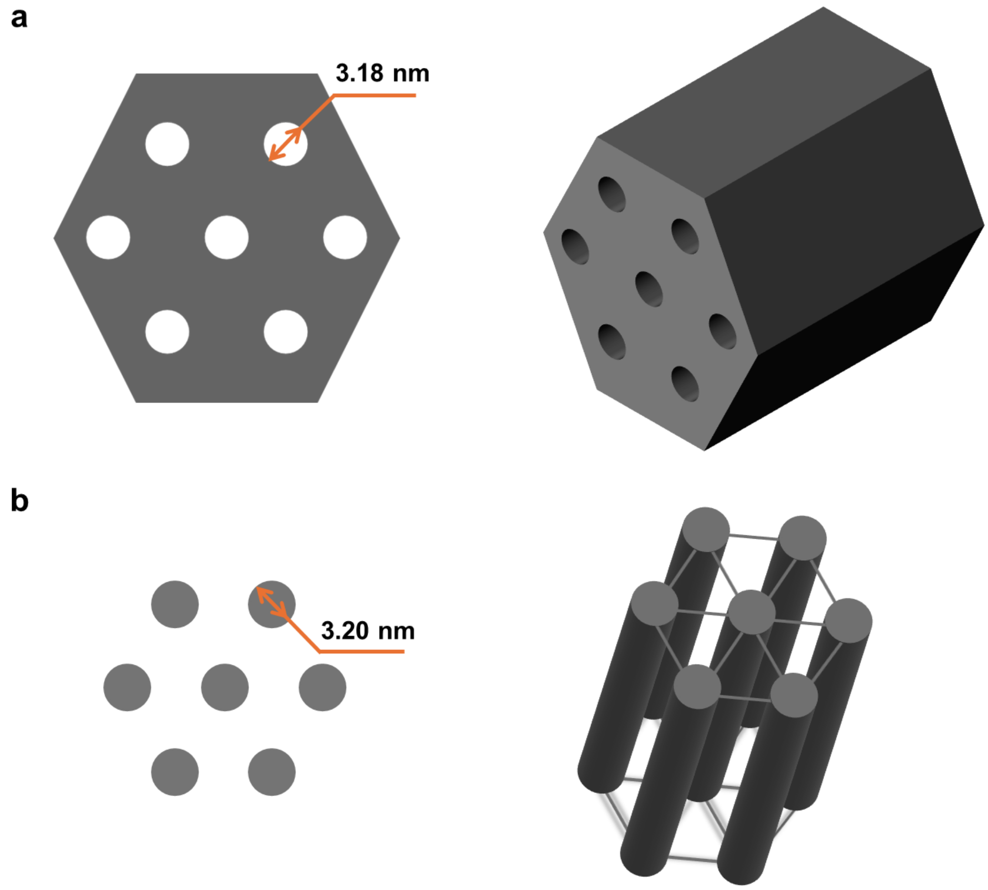

Figure S5. (a, b) Scheme showing the structure of concave-FePc (a) and convex-FePc (b).

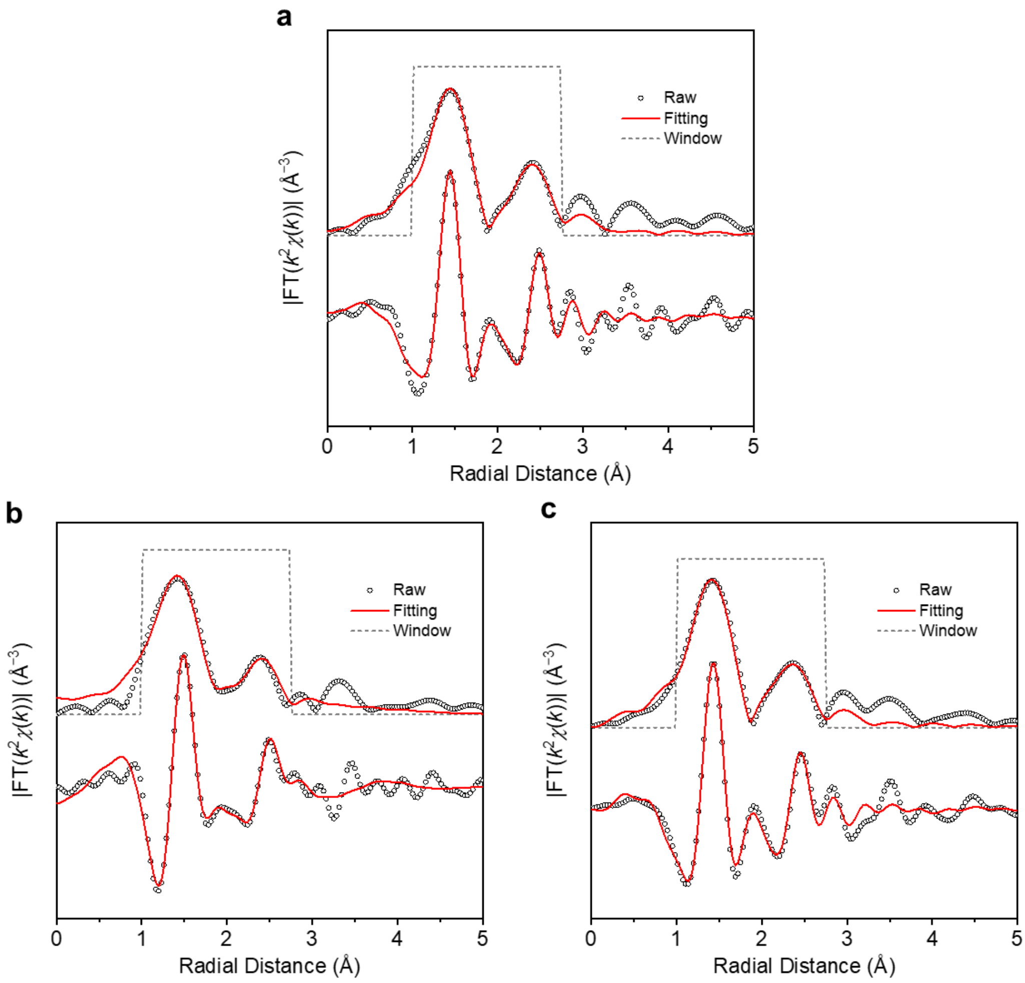

Figure S6. (a-c) EXAFS fitting of FePc (a), convex-FePc (b), and concave-FePc (c).

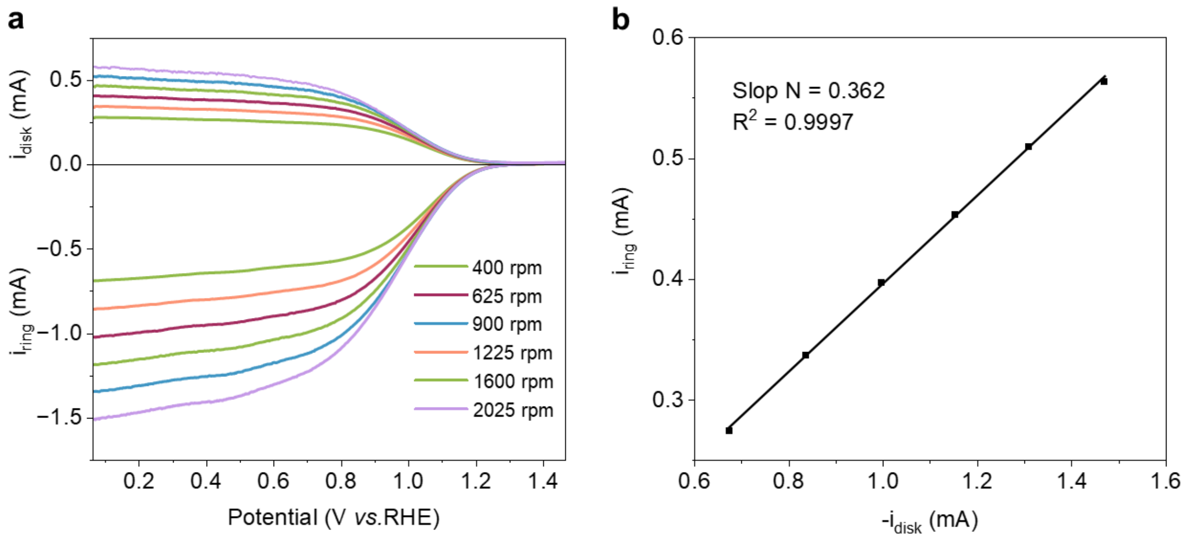

Figure S7. (a, b) Determination of collection efficiency of the ring electrode via a one-electron $Fe(CN)_6^{4-}/Fe(CN)_6^{3-}$ redox couple.

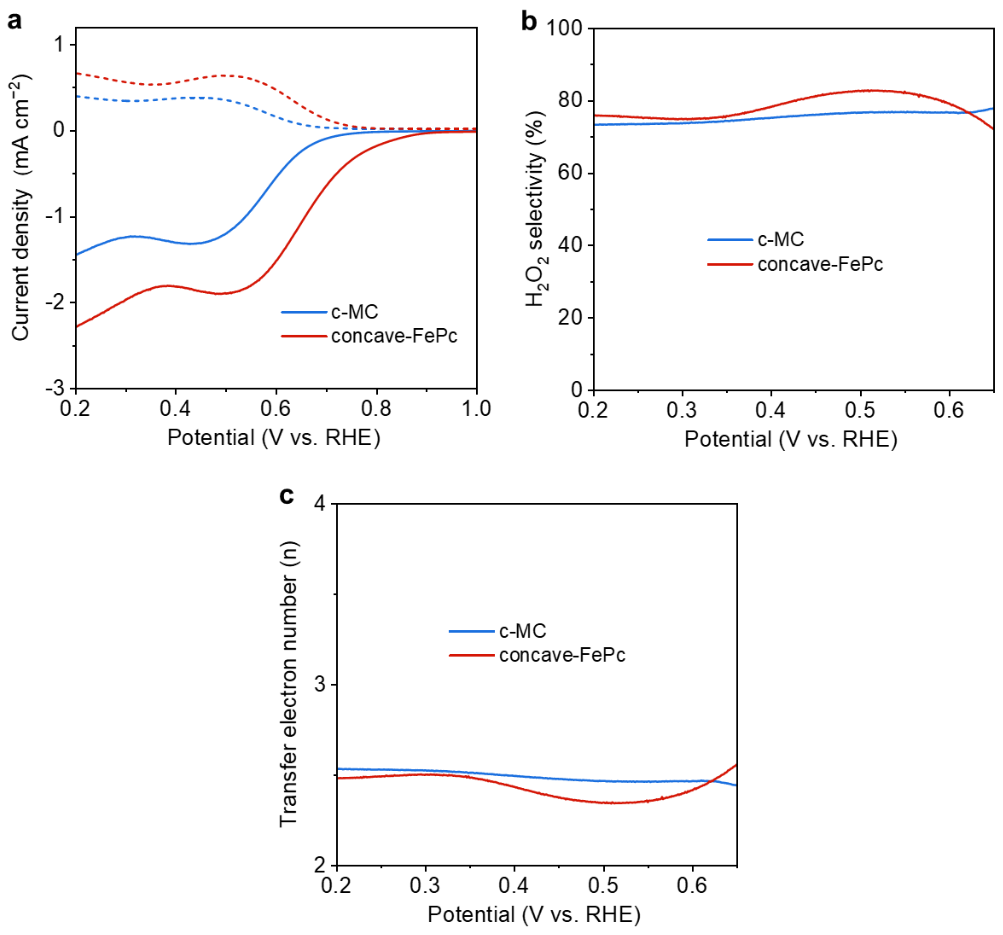

Figure S8. (a) ORR LSV curves recorded on the disk electrode (bottom panel) and H_2O_2 oxidation LSV at the ring electrode (upper panel) at 1600 rpm, (b) H_2O_2 selectivity, and (c) Number of transferred electrons (n) for c-MC and concave-FePc.

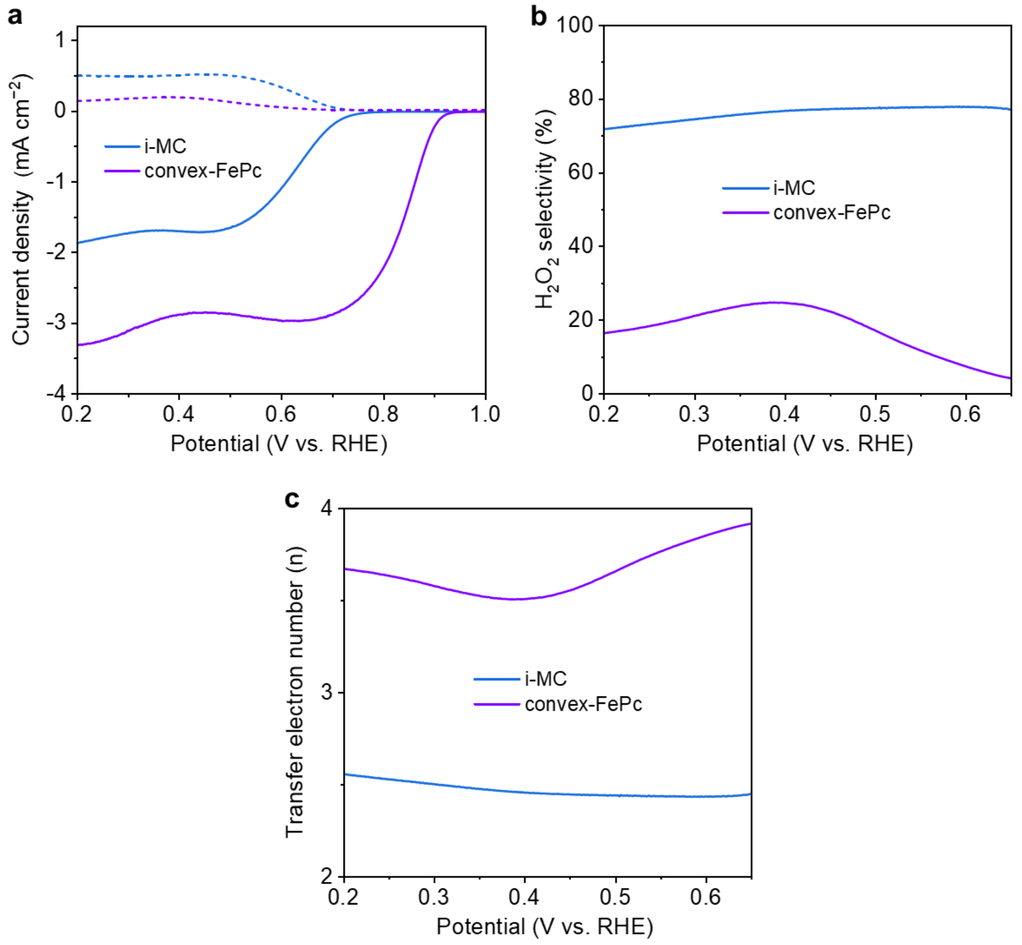

Figure S9. (a) ORR LSV curves recorded on the disk electrode (bottom panel) and H_2O_2 oxidation LSV at the ring electrode (upper panel) at 1600 rpm, (b) H_2O_2 selectivity, and (c) Number of transferred electrons (n) for i-MC and convex-FePc.

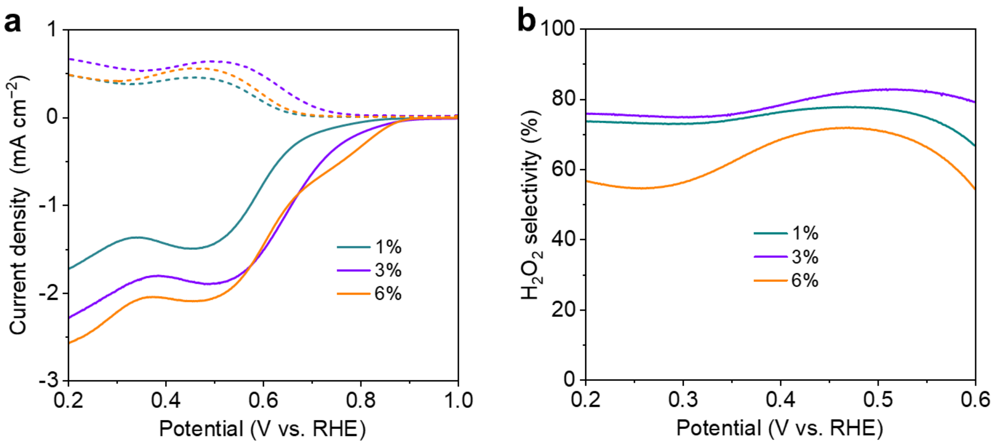

Figure S10. Electrocatalytic ORR performance of concave-FePc prepared with different feeding mass ratios of FePc:c-MC from 1 wt%, 3 wt%, to 6 wt%, labeled as 1%, 3%, and 6%, respectively. (a) ORR LSV curves recorded on the disk electrode (bottom panel) and H_2O_2 oxidation LSV at the ring electrode (upper panel) at 1600 rpm. (b) H_2O_2 selectivity.

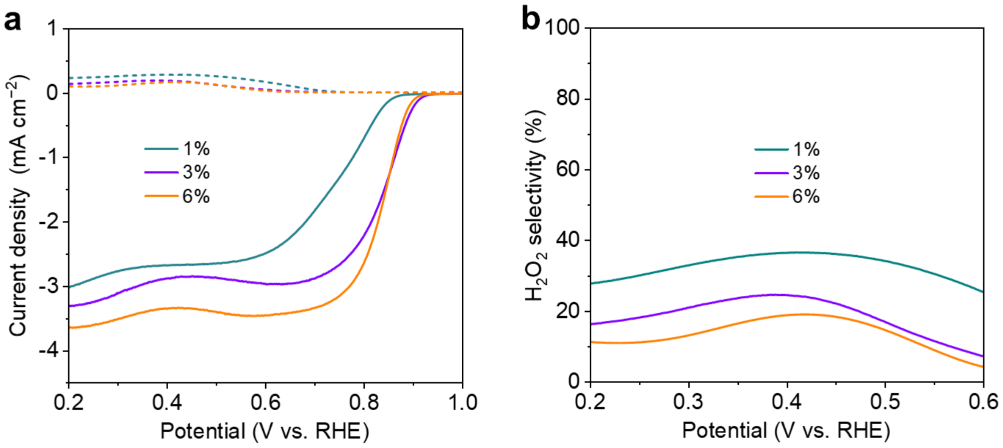

Figure S11. Electrocatalytic ORR performance of convex-FePc prepared with different feeding mass ratios of FePc:i-MC support from 1 wt%, 3 wt%, to 6 wt%, labeled as 1%, 3%, and 6%, respectively. (a) ORR LSV curves recorded on the disk electrode (bottom panel) and H_2O_2 oxidation LSV at the ring electrode (upper panel) at 1600 rpm. (b) H_2O_2 selectivity.

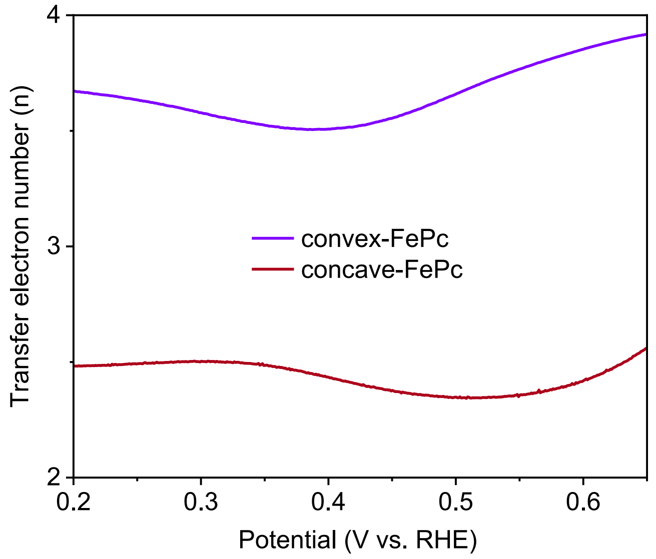

Figure S12. Number of transferred electrons (n) of convex-FePc and concave-FePc in ORR.

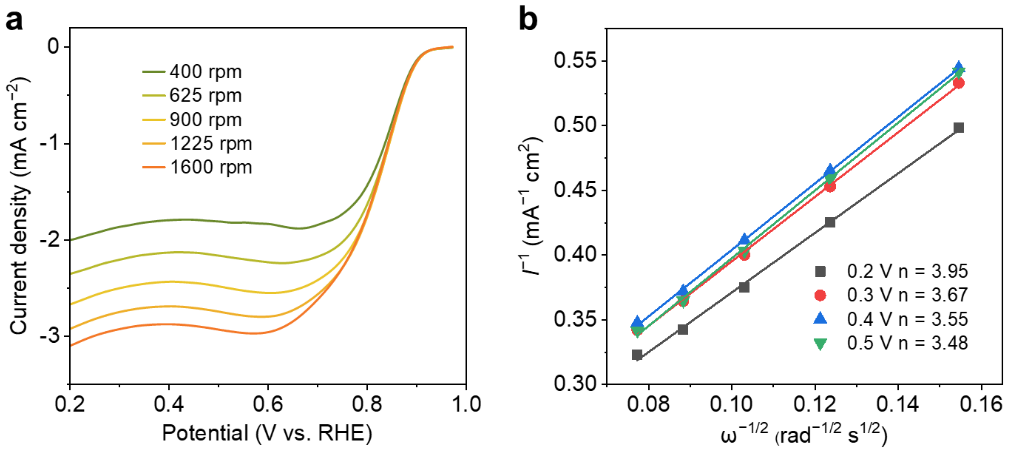

Figure S13. (a) LSV curves on convex-FePc at various RDE speeds. (b) K-L plots at different potentials.

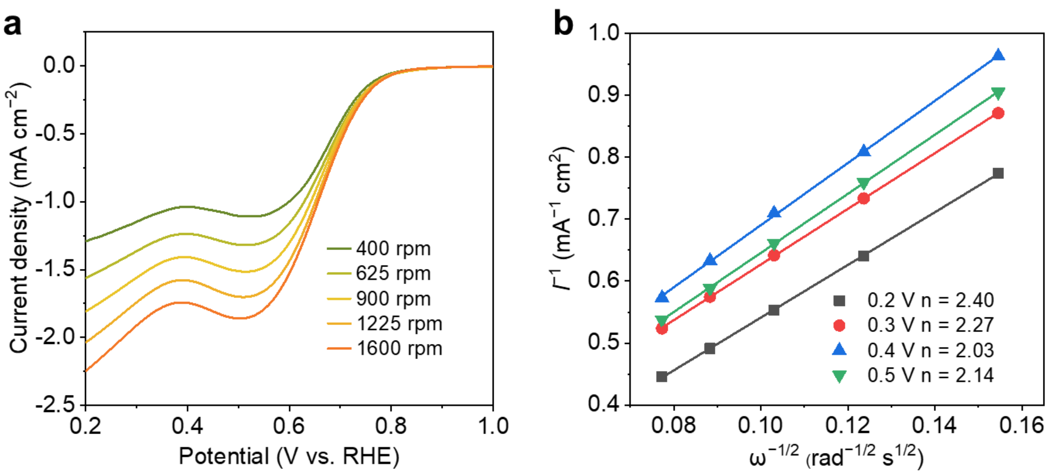

Figure S14. (a) LSV curves on concave-FePc at various RDE speeds. (b) K-L plots at different potentials.

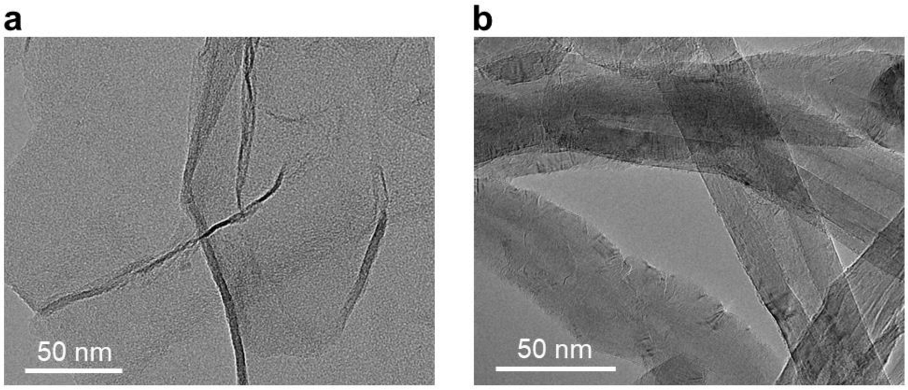

Figure S15. TEM images for FePc/graphene (a) and FePc/carbon nanotube (b).

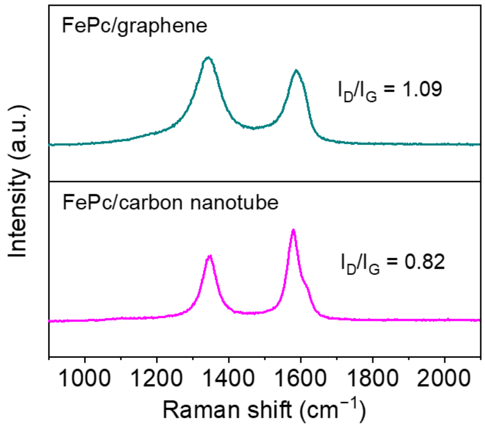

Figure S16. Raman spectra for FePc/graphene and FePc/carbon nanotube.

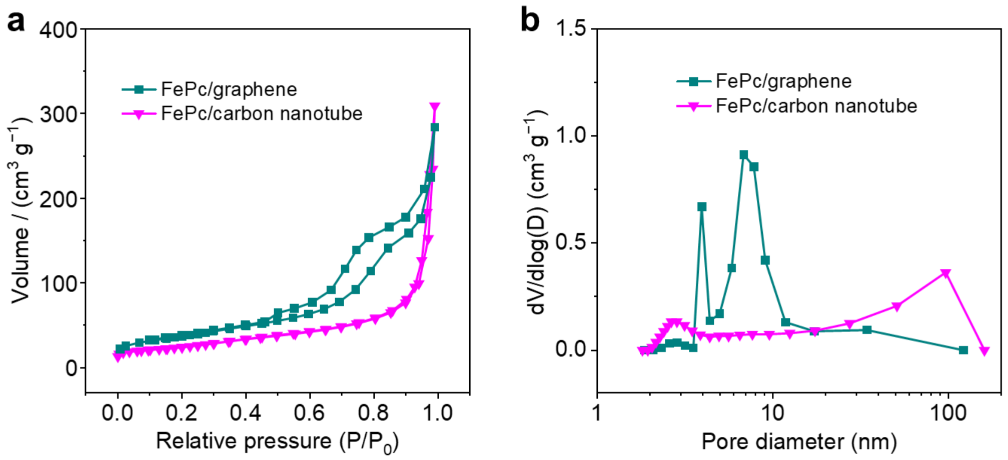

Figure S17. (a) N_2 adsorption-desorption isotherms and (b) pore size distributions for FePc/graphene and FePc/carbon nanotube.

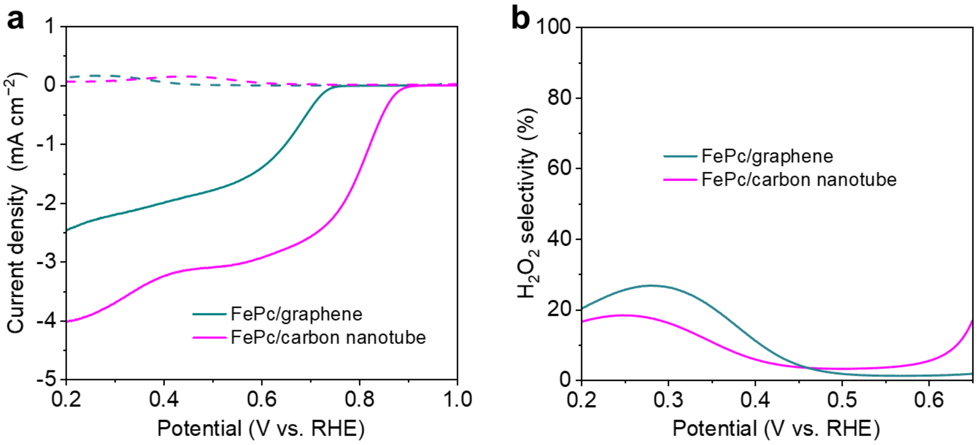

Figure S18. (a) ORR LSV curves recorded on the disk electrode (bottom panel) and H_2O_2 oxidation LSV at the ring electrode (upper panel) at 1600 rpm, and (b) corresponding H_2O_2 FEs for FePc/graphene and FePc/carbon nanotube.

Two reference catalysts were prepared by loading FePc on graphene (2D nanosheet) and carbon nanotube (diameter of around 40-50 nm) (Figure S15), named as FePc/graphene and FePc/carbon nanotube, respectively. The two carbon supports can be considered as a planar surface due to the negligible local curvature of 2D graphene and large-diameter carbon nanotube. Raman spectra show that FePc/carbon nanotube has less defect than FePc/graphene (Figure S16). BET surface areas are 132.8 and 86.9 m^2 g^{-1} for FePc/graphene and FePc/carbon nanotube, respectively. The pore size distribution shows different pore feature (Figure S17). FePc/graphene has mesopores less than 10 nm. FePc/carbon nanotube has fewer mesopores below 10 nm, while the 1D nanotube can reticulate macroporous network with pore size of around 100 nm. In O_2 reduction, the two catalysts exhibit high selectivity for $4e^-$ pathways with H_2O_2 selectivity less than 30% (Figure S18), Compared to FePc/carbon nanotube, FePc/graphene shows lower current density, probably due to the stacking of 2D graphene that limits mass transport. These results suggest that surface area, pore distribution, and defect degree are not main factors controlling ORR selectivity.

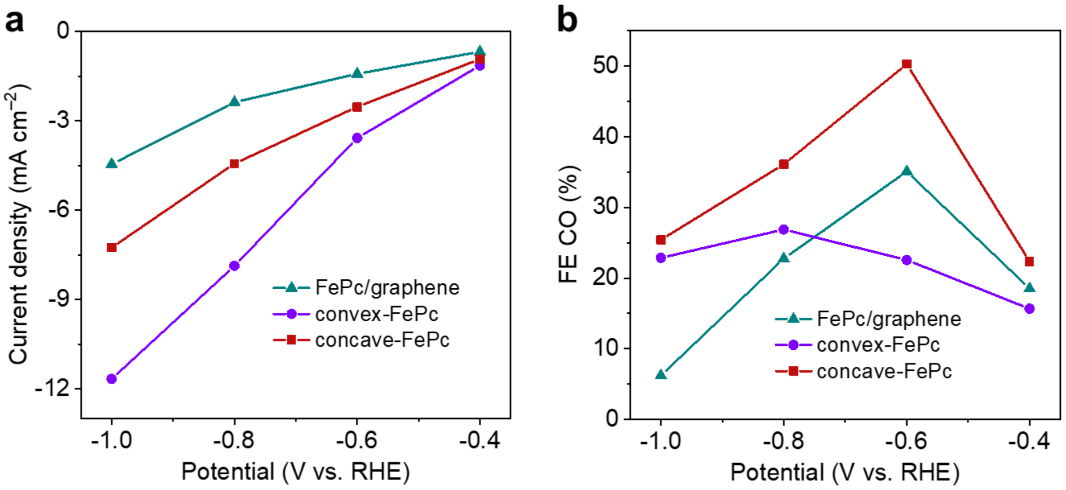

Figure S19. (a) Total current densities and (b) CO FEs in CO_2 reduction on various catalysts.

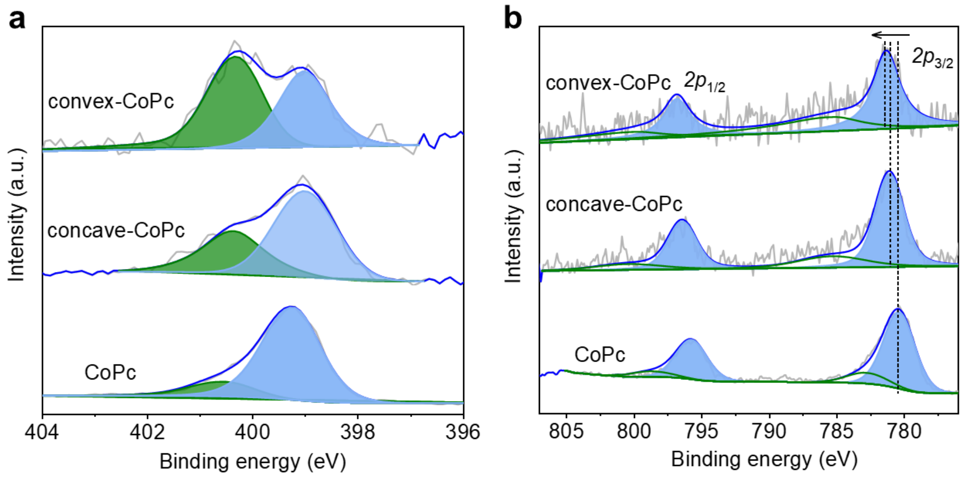

Figure S20. (a) XPS N 1s and (b) Co 2p spectra of CoPc, concave-CoPc, and convex-CoPc.

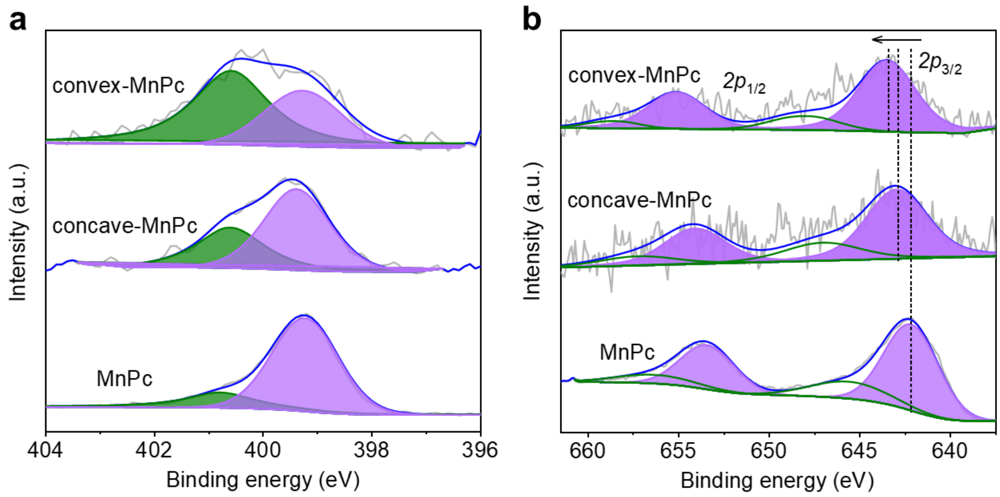

Figure S21. (a) XPS N 1s and (b) Mn 2p spectra of MnPc, concave-MnPc, and convex-MnPc.

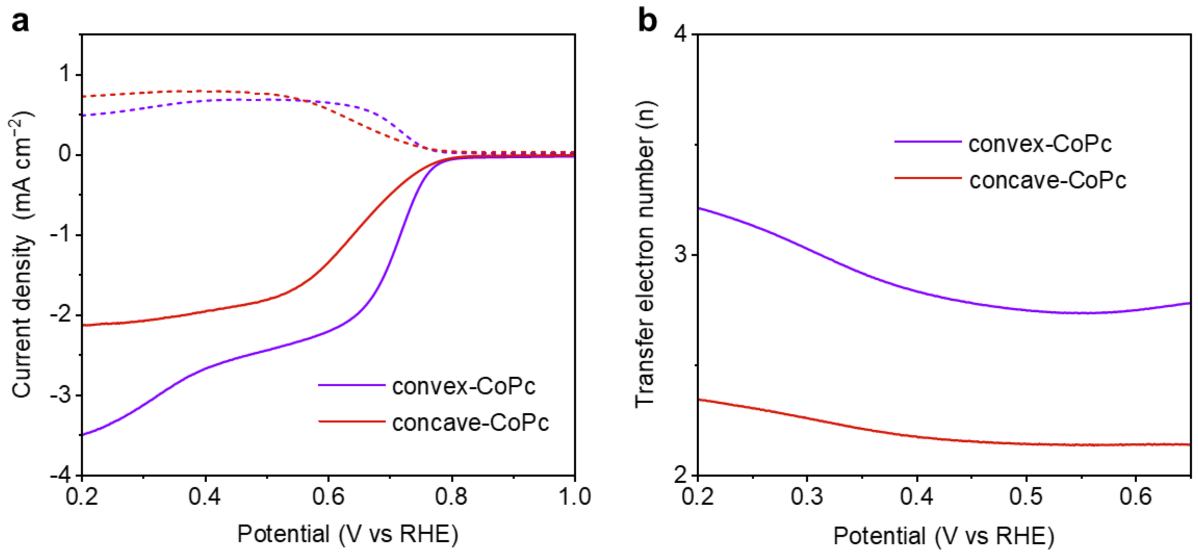

Figure S22. (a) LSVs recorded on the disk electrode (bottom panel) and the simultaneous H_2O_2 oxidization current densities at the ring electrode (upper panel) at 1600 rpm for convex-CoPc and concave-CoPc. (b) Number of transferred electrons (n) of convex-CoPc and concave-CoPc in ORR.

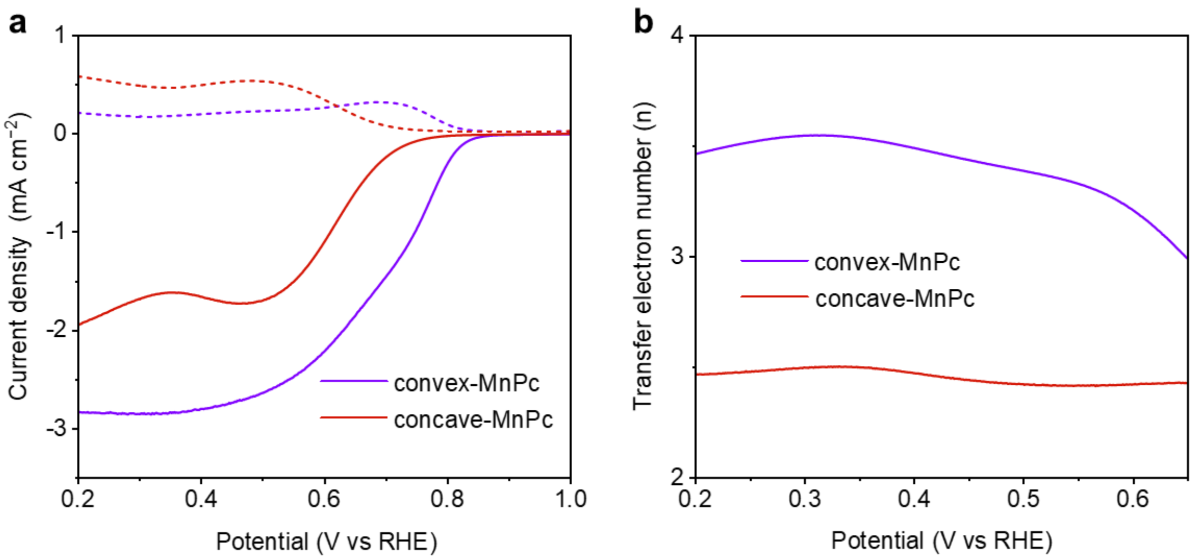

Figure S23. (a) LSVs recorded on the disk electrode (bottom panel) and the simultaneous H_2O_2 oxidization current densities at the ring electrode (upper panel) at 1600 rpm for convex-MnPc and concave-MnPc. (b) Number of transferred electrons (n) of convex-MnPc and concave-MnPc in ORR.

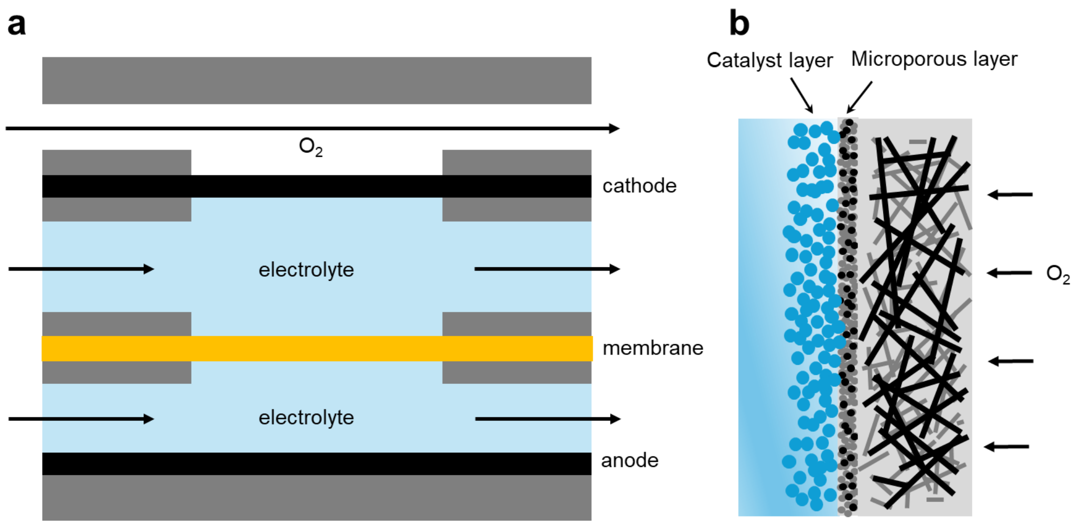

Figure S24. (a) Schematic showing a flow-cell electrolyzer used for large-current H_2O_2 synthesis. (b) Structure of gas-diffusion electrode.

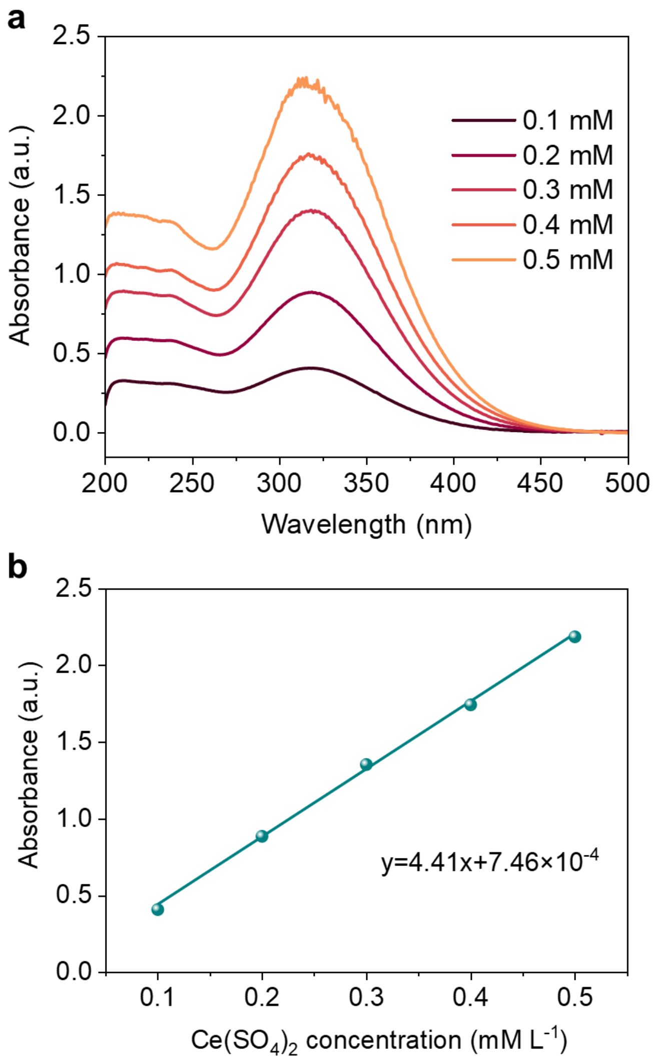

Figure S25. (a) UV-Vis absorption spectra of standard cerium titration solutions with an incremental concentration. (b) Linear fitting plot of absorbance at 318 nm as a function of Ce^{4+} concentration.

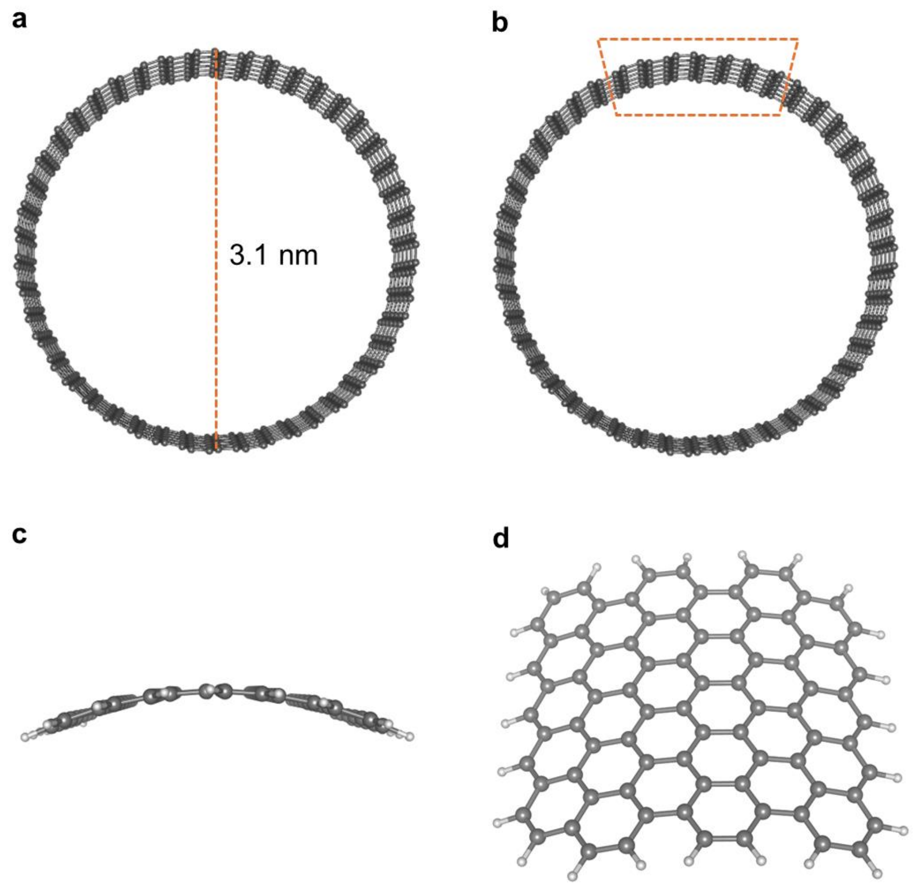

Figure S26. (a) A single-wall carbon nanotube with similar diameter to cylindrical mesopore of c-MC. (b) Labeled area showing the representative curved support. (c, d) Curved carbon terminated by H atoms used to support FePc.

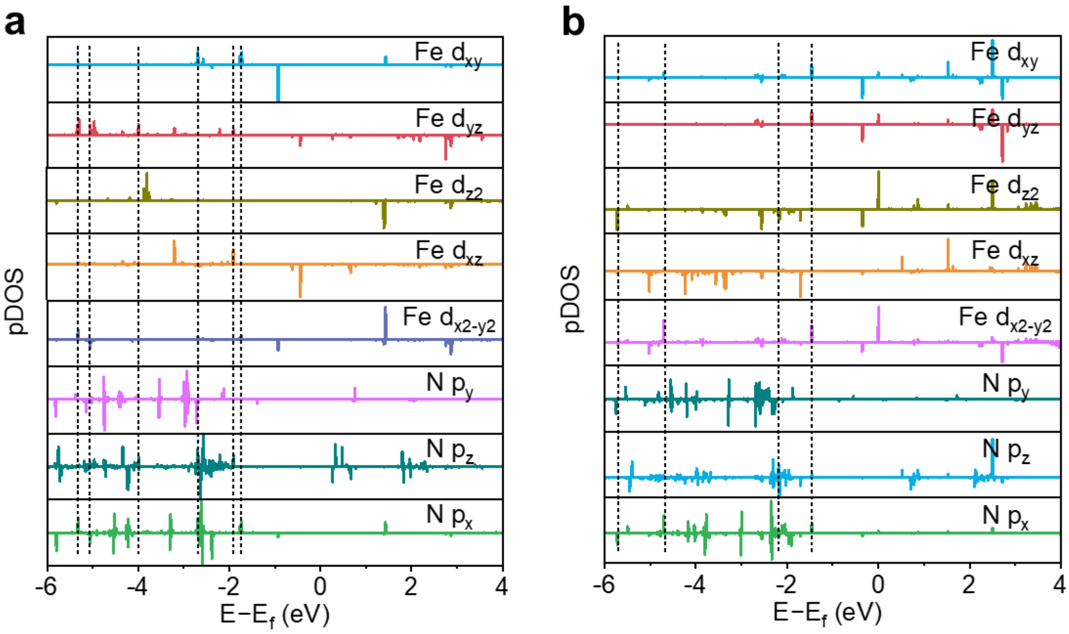

Figure S27. (a, b) Calculated projected density of states showing the orbital interaction between the Fe 3d and N 2p orbitals for convex-FePc (a) and concave-FePc (b).

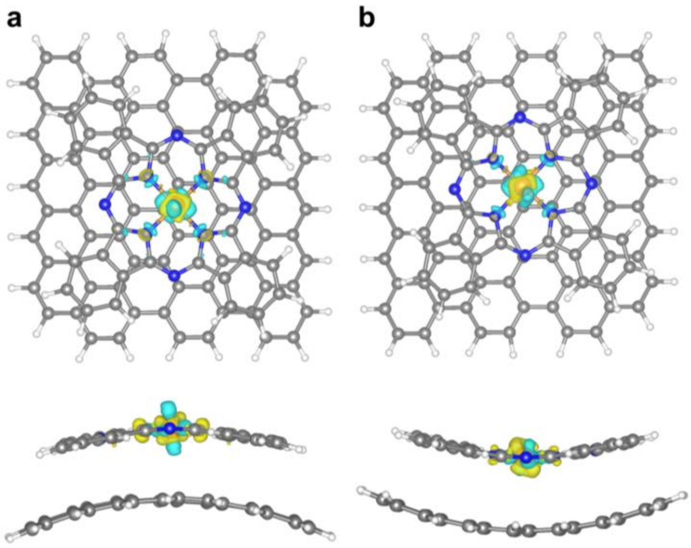

Figure S28. Charge density difference analysis for convex-FePc (a) and concave-FePc (b). Yellow and cyan isosurfaces indicate the electron accumulation and depletion. The gray, blue, orange, and white balls represent C, N, Fe, and H atoms, respectively.

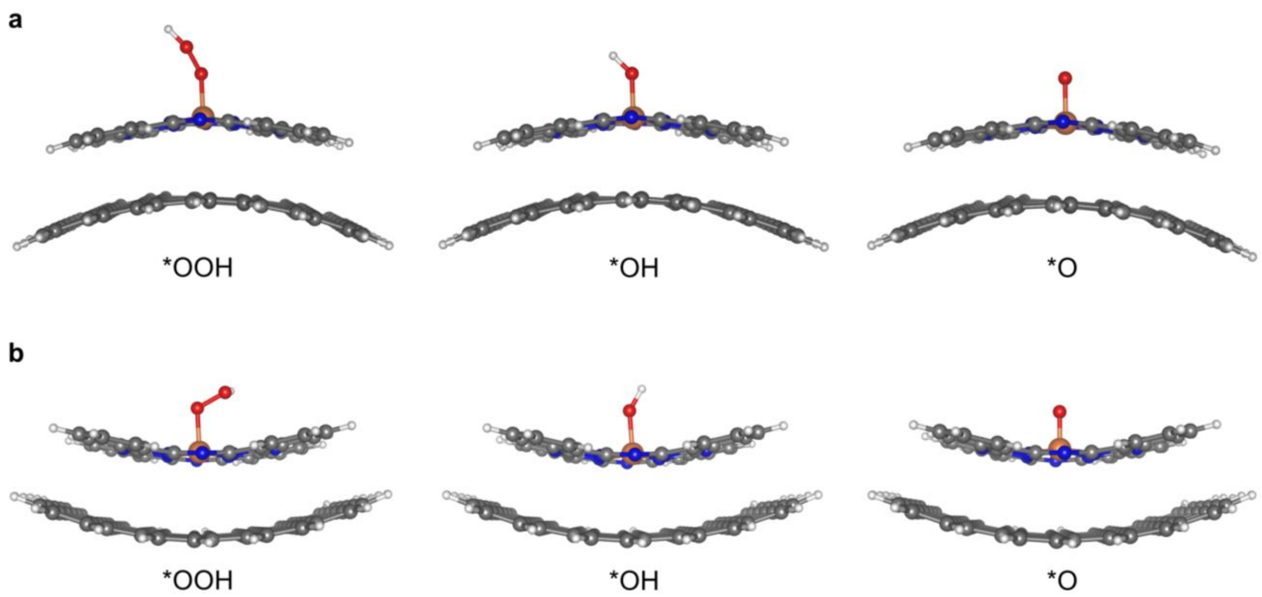

Figure S29. (a, b) Optimized adsorption configurations of *OOH, *O, and *OH intermediates on convex-FePc (a) and concave-FePc (b). The gray, blue, orange, red, and white balls represent C, N, Fe, O, and H atoms, respectively.

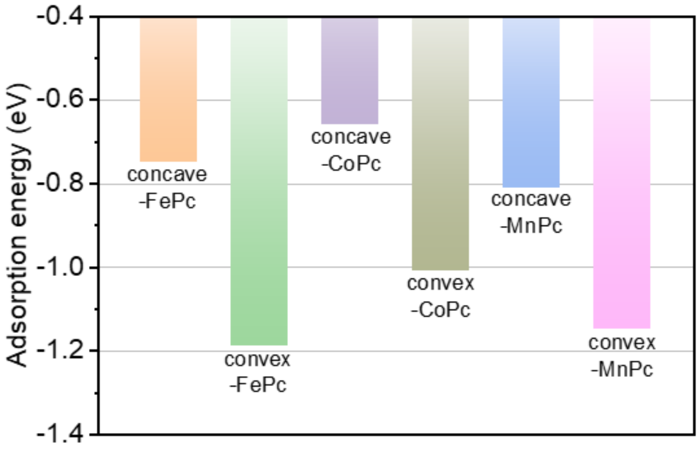

Figure S30. Adsorption energy of *OOH on convex-MPc and concave-MPc.

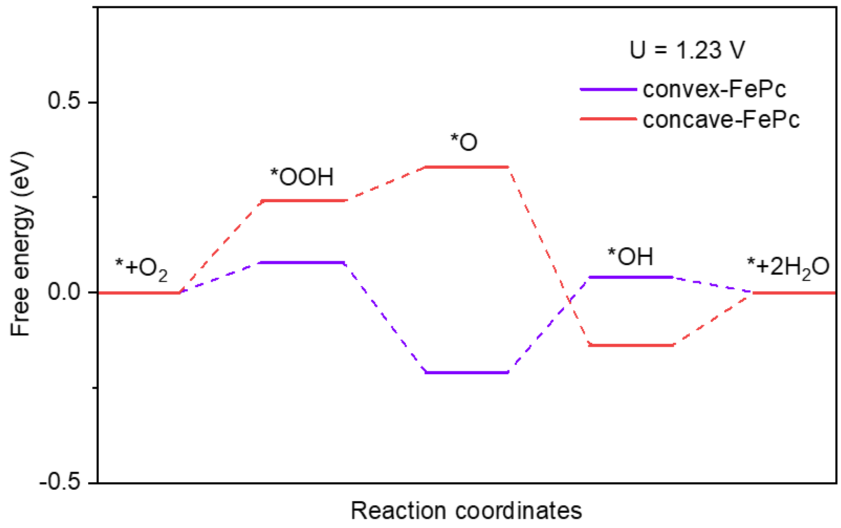

Figure S31. Gibbs free energy diagrams for 4e^{-} ORR pathways at U = 1.23 V.

Table S1. Summary of elemental contents of catalysts.

Catalysts	N content by XPS (at%)	Fe content by XPS (at%)	Fe:N ratio by XPS	Fe loading by ICP-MS (wt%)	FePc:carbon mass ratio by ICP
convex-FePc	3.15	0.41	7.68	0.22	1:43.6
concave-FePc	2.55	0.32	7.96	0.15	1:64.4

Table S2. Structural parameters of catalysts obtained by EXAFS fitting. There are the average coordination number (N), path distance (R), Debye-Waller factor (σ^2), threshold energy correction (ΔE), and the R-Factor of the fitting.

Catalysts	Shell	N	R(Å)	σ^2(Å^2)	ΔE(eV)	R-Factor
FePc	Fe−N	3.92	1.96	0.007	3.373	0.008
	Fe−C	8.28	2.97	0.005		
convex-FePc	Fe−N	4.32	1.95	0.011	-5.624	0.015
	Fe−C	7.67	2.95	0.012		
Concave-FePc	Fe−N	4.16	1.95	0.007	4.148	0.004
	Fe−C	7.83	2.94	0.006		

References

1. Marcano, D. C.; Kosynkin, D. V.; Berlin, J. M.; Sinitskii, A.; Sun, Z.; Slesarev, A.; Alemany, L. B.; Lu, W.; Tour, J. M., Improved synthesis of graphene oxide. *ACS Nano* **2010**, *4*, 4806-4814.

2. Gu, Y.; Tan, Y.; Tan, H.; Han, Y.; Cheng, D.; Lin, F.; Qian, Z.; Zeng, L.; Zhang, S.; Zeng, R.; Liu, Y.; Guo, H.; Luo, M.; Guo, S., Industrial electrosynthesis of hydrogen peroxide over p-block metal single sites. *Nat. Synth.* **2025**, *4*, 614-621.

3. Kresse, G.; Furthmüller, J., Efficiency of ab-initio total energy calculations for metals and semiconductors using a plane-wave basis set. *Comp. Mater. Sci.* **1996**, *6*, 15-50.

4. Kresse, G.; Furthmüller, J., Efficient iterative schemes for ab initio total-energy calculations using a plane-wave basis set. *Phys. Rev. B Condens. Matter* **1996**, *54*, 11169-11186.
